# Light-Responsive and Dual-Targeting Liposomes: From Mechanisms to Targeting Strategies

**DOI:** 10.3390/molecules29030636

**Published:** 2024-01-30

**Authors:** Ahmed M. Agiba, José Luis Arreola-Ramírez, Verónica Carbajal, Patricia Segura-Medina

**Affiliations:** 1 Escuela de Ingeniería y Ciencias, Tecnológico de Monterrey, Monterrey 64849, Mexico; ahmed.agiba@tec.mx; 2 Departamento de Investigación en Hiperreactividad Bronquial, Instituto Nacional de Enfermedades Respiratorias Ismael Cosío Villegas, Calzada de Tlalpan 4502, Mexico City 14080, Mexico; arreolaj2002@yahoo.com.mx (J.L.A.-R.); estelacs@hotmail.com (V.C.); 3 Escuela de Medicina y Ciencias de la Salud, Tecnológico de Monterrey, Mexico City 14380, Mexico

**Keywords:** smart nanocarriers, light-responsive liposomes, dual-targeted stimuli-responsive liposomes, photo-triggered targeting strategies, light-triggering mechanisms, NIR-responsive nanocarriers, current challenges, future perspectives

## Abstract

In recent years, nanocarriers have played an ever-increasing role in clinical and biomedical applications owing to their unique physicochemical properties and surface functionalities. Lately, much effort has been directed towards the development of smart, stimuli-responsive nanocarriers that are capable of releasing their cargos in response to specific stimuli. These intelligent-responsive nanocarriers can be further surface-functionalized so as to achieve active tumor targeting in a sequential manner, which can be simply modulated by the stimuli. By applying this methodological approach, these intelligent-responsive nanocarriers can be directed to different target-specific organs, tissues, or cells and exhibit on-demand controlled drug release that may enhance therapeutic effectiveness and reduce systemic toxicity. Light, an external stimulus, is one of the most promising triggers for use in nanomedicine to stimulate on-demand drug release from nanocarriers. Light-triggered drug release can be achieved through light irradiation at different wavelengths, either in the UV, visible, or even NIR region, depending on the photophysical properties of the photo-responsive molecule embedded in the nanocarrier system, the structural characteristics, and the material composition of the nanocarrier system. In this review, we highlighted the emerging functional role of light in nanocarriers, with an emphasis on light-responsive liposomes and dual-targeted stimuli-responsive liposomes. Moreover, we provided the most up-to-date photo-triggered targeting strategies and mechanisms of light-triggered drug release from liposomes and NIR-responsive nanocarriers. Lastly, we addressed the current challenges, advances, and future perspectives for the deployment of light-responsive liposomes in targeted drug delivery and therapy.

## 1. Introduction

### 1.1. Liposomes as Drug Nanocarriers

**Figure 1:**
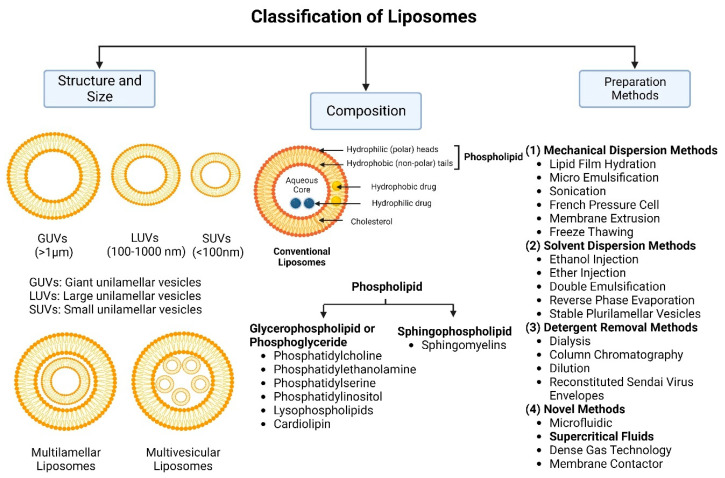
Classification of liposomes according to their structures, sizes, compositions, and preparation methods.

Nanocarriers were first discovered in the early 1960s, when scientists proposed the application of liposomes for drug delivery [1]. Since then, many nanocarrier systems have been developed and approved for marketing by the U.S. Food and Drug Administration (FDA) and the European Medicines Agency (EMA). Nanocarriers have been extensively used in drug delivery owing to their exceptional physicochemical properties, such as nanometric particle size, surface charge, high entrapment efficiency, and drug loading capacity [2]. Nanocarriers used in cancer treatment have received particular attention from researchers worldwide, since most conventional chemotherapeutic drugs cause systemic toxicity resulting from their poor stability in biological systems, non-selectivity, and non-specificity toward cells expressing the targeted receptors [3]. The first evidence on the feasibility and effective use of nanocarriers in cancer treatment was reported in 1976 by Langer et al. [4], who prepared the first sustained-release, long-circulating poly(ethylene glycol)-poly(lactic acid-ethanolic acid) (PEG-PLEA) nanoparticles, which were later approved by the FDA as the first nanomedicine for therapeutic use in cancer treatment. Generally, nanocarriers are categorized into two main classes: organic and inorganic nanocarriers [5,6]. Organic nanocarriers include nanoemulsions (10–1000 nm), nanosuspensions (<1 µm), nanoliposomes (50–450 nm), polymeric nanoparticles (10 nm to 1 µm), solid-lipid nanoparticles (10–1000 nm), and nanodendrimers (15–200 nm), while inorganic nanocarriers include gold nanoparticles (5–400 nm), silver nanoparticles (1–100 nm), mesoporous silica nanoparticles (30–300 nm), and superparamagnetic iron oxide nanoparticles (100 nm to 5 µm). These tumor-targeting, nano-sized drug delivery systems were developed primarily to reduce the systemic toxicity of chemotherapeutic drugs through encapsulation into nanocarrier systems, which allowed for site-specific delivery with improved passive and active drug targeting (i.e., disease-specific targeted therapeutics). Of all these nanocarriers, liposomes are very promising drug delivery systems with the advantages of being non-toxic, biocompatible, and biodegradable [2]. Liposomes were first discovered by Bangham et al. [7] in 1964. They discovered how membrane molecules interact with water to form unique structural forms, which were described as swollen phospholipid systems [7]. Briefly, liposomes are defined as vesicular systems consisting of one or more concentric spheres of phospholipid bilayers separated by aqueous or buffer compartments [7,8]. When phospholipids are dispersed in an aqueous medium like water or buffer, the hydration of phospholipid polar heads results in a heterogeneous mixture of spherical structures, generally referred to as vesicles, most of which contain multiple phospholipid bilayers forming concentric spherical shells [7,8]. Those were the liposomes first reported by Bangham et al. [1,7,8], nowadays referred to as multilamellar large vesicles (MLVs). The sonication of these lipid dispersions results in the size reduction of these liposomes to vesicles containing only a single bilayer with diameters ranging from 20 to 100 nm, later referred to as small unilamellar vesicles (SUVs) [9]. Large unilamellar vesicles (LUVs, 100–1000 nm) are intermediate in size between MLVs (>700 nm) and SUVs [9]. The main components of liposomes are phospholipids and cholesterol, which are naturally occurring substances [9,10]. Phospholipids are amphiphilic molecules with hydrophobic non-polar tails and hydrophilic polar heads. These amphiphilic molecules spontaneously organize into liposomes in an aqueous or buffer environment, driven by hydrophobic interactions and other intermolecular interactions [11]. The proper choice of phospholipid is important to achieve the desired effects. Table 1 shows the most commonly used phospholipids in the preparation of liposomes (data extracted from the Sigma-Aldrich (Burlington, MA, USA) and Avanti Polar Lipids (Alabaster, AL, USA) databases). Figure 1 shows the classification of liposomes according to their structures, sizes, compositions, and preparation methods.

Phospholipids play key roles in the stability of liposomes in the systemic circulation, liposomal encapsulation, drug loading efficiency, and drug release at target sites. For instance, the use of 1,2-distearoyl-*sn*-glycero-3-phosphocholine (DSPC) in the preparation of liposomes resulted in a higher drug encapsulation efficiency compared to 1-palmitoyl-2-oleoyl-*sn*-glycero-3-phosphocholine (POPC) and 1,2-dipalmitoyl-*sn*-glycero-3-phosphocholine (DPPC). This was mainly due to the lengthy fatty acid chain of DSPC as well as the rigidity of the acyl chains of DSPC [12]. Furthermore, the use of *n-*(methoxypolyethylene glycol 5000 carbamoyl)-1,2-dipalmitoyl-*sn-*glycero-3-phosphatidylethanolamine, monosodium salt (MPEG-5000-DPPE-Na) prolonged the blood circulation time of liposomes, owing to the additional steric hindrance of MPEG-5000-DPPE-Na, which reduced the liposomal uptake by the reticuloendothelial system (RES) [13]. Additionally, 1,2-dipalmitoyl-*sn*-glycero-3-phosphoglycerol, sodium salt (DPPG-Na) exhibited fusogenic activity that improved the ability of liposomes to cross the cell membrane [14]. In general, liposomes can enter cells either by endocytosis (i.e., the process of capturing liposomes from outside by engulfing them with the cell membrane) or via exocytosis membrane fusion (i.e., the process where two phospholipid bilayers merge into a single continuous bilayer). Anionic liposomes showed faster endocytosis that enhanced their intracellular uptake, while fusogenic liposomes demonstrated an ability to fuse and penetrate the cell membrane [2,9]. Fusogenic liposomes are a particular type of liposome that are capable of causing fusion with biological membranes, thereby improving cell-type-specific delivery and therapeutic efficacy. They are mainly composed of phospholipids, such as dioleoyl-phosphatidylethanolamine (DOPE) and cholesteryl hemisuccinate (CHEMS) [15]. On the other hand, the use of cholesterol in liposomes aims to provide additional rigidity to the bilayer system in order to enhance liposome stability by increasing the molecular packaging of phospholipid molecules, prompting drug retention inside the bilayer system, and reducing the permeability of phospholipid bilayers [16]. In fact, cholesterol does not form bilayers by itself but will dissolve readily in the phospholipid–water bilayer system [10]. The unique feature of liposomes is their ability to compartmentalize and encapsulate both hydrophilic and hydrophobic drugs. This unique feature, along with biodegradability, biocompatibility, safety, non-toxicity, and targetability, made liposomes very attractive nanocarriers to maximize drug delivery and activity [2,9].

**Figure 2:**
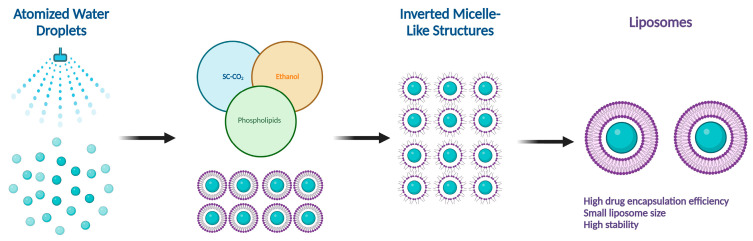
Supercritical CO_2_ (SC-CO_2_)-assisted liposome formation.

There are several methods for the preparation of liposomes (Figure 1) [2,9], such as thin-film hydration (or thin-layer evaporation), reverse-phase evaporation, double emulsification, ether injection, ethanol injection, and detergent removal. However, all these techniques are used for lab-scale production and mostly require the use of organic solvents in high concentrations and ratios. Moreover, they exhibit difficulty in controlling size and intercalation efficiency. Supercritical carbon dioxide (SC-CO_2_) (Figure 2) is a novel technique suitable for the large-scale production of liposomes with the advantages of high encapsulation efficiency and uniform particle size distribution without the necessity of post-formation processes such as sonication or extrusion [17,18]. In SC-CO_2_, CO_2_ is premixed with lipids and then enters a chamber with atomized water droplets. As a result of the high diffusion ability of CO_2_ and the reduced viscosity of the solution, lipids would coat water droplets at higher rates, resulting in inverted micelle-like structures, which are further stabilized by another layer of lipids placed at the bottom of the chamber [19].

Numerous liposomal formulations have been clinically approved for human use to treat cancer and other chronic diseases, and are currently available on the global pharmaceutical market. Table 2 shows FDA- and EMA-approved liposomal drug formulations [20,21]. The Orange Book identifies drug products approved on the basis of safety and effectiveness by the FDA [20]. The electronic medicines compendium (EMC) identifies drug products approved for human use in the UK and Europe [21].

### 1.2. Targeting Mechanisms of Liposomes

The tumor-targeted delivery of liposomes can be achieved by two main targeting mechanisms: passive and active targeting.

#### 1.2.1. Passive Targeting of Liposomes

In the passive targeting mechanism, liposomes are transported through the tumor interstitium to the target cells through capillary fenestrations and channels through passive diffusion or convection [22]. The tumor angiogenesis induces irregularities in endothelial cells with different pore sizes, ranging from 100 nm to 2 μm [23]. The differences in pore sizes and size distributions between the tumor microvasculature of endothelial cells and the tighter structures of normal cells make liposomes more easily accessible to the cancerous sites. Additionally, liposomes exploit the enhanced permeability and retention (EPR) effect for tumor targeting by improving the amounts of drugs delivered to tumor sites [23]. In order to passively target liposomes to tumor cells, liposomes should possess some physical and structural characteristics, such as the following: (1) the size of the liposomes should be in the range of 10–100 nm; (2) they should carry a neutral or anionic charge in order to avoid renal elimination; and (3) they should be protected from the RES [22,24].

#### 1.2.2. Active Targeting of Liposomes

**Figure 3:**
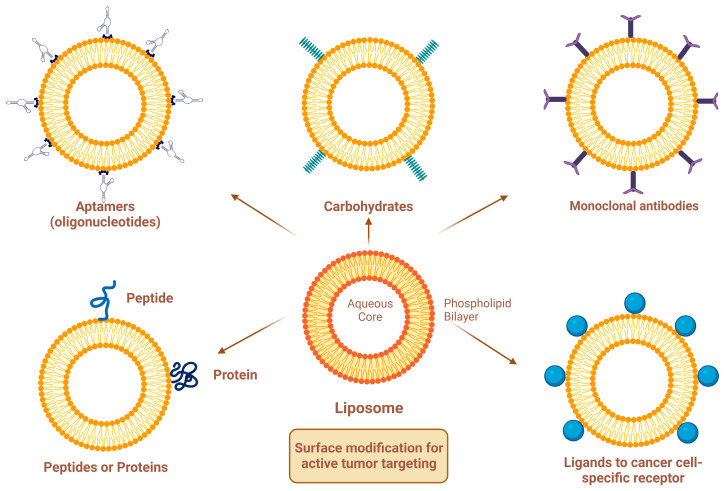
Surface modification of liposomes for active targeting.

**Figure 4:**
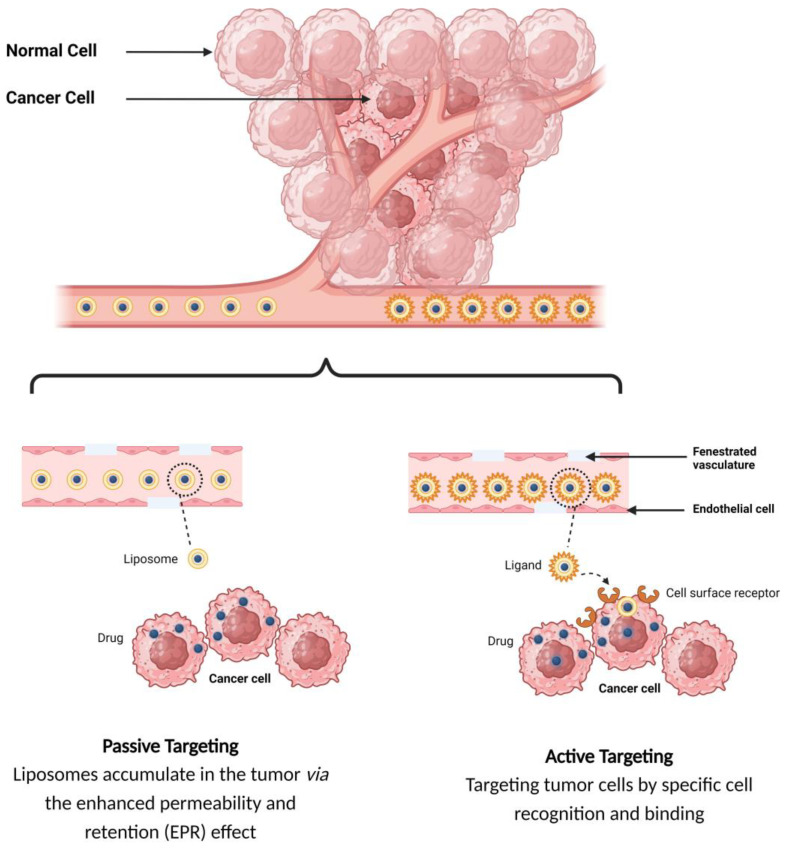
Targeting mechanisms of liposomes.

Site-specific drug delivery is a method of targeting drugs to specific sites in a manner that increases their therapeutic indexes and reduces their possible side effects and toxicities [22,24]. Liposomes can reach tumor sites passively through the EPR effect [23], while surface-modified (or surface-engineered) liposomes act by binding to specific receptors overexpressed by cancer cells, such as epidermal growth factor receptor (EGFR), folate receptor (FR), transferrin receptor (TFR), and other receptors (Figure 3) [22]. Since targeting the overexpressed surface receptors of cancer cells in order to enhance cellular uptake and intracellular activity is a promising approach, several cell surface strategies have been developed so far, aiming towards achieving the targeted inhibition of these receptors [22,24]. The efficiency of active targeting and ligand receptor interaction is dependent on certain factors [25], such as (1) the extent of receptor expression level on tumor cells relative to non-tumor cells, (2) the availability of surface receptors on tumor cells, (3) the internalization rate, and (4) the heterogeneity of receptor expression in tumor cells. Active targeting can be achieved through the surface engineering of liposomes via decoration with aptamers (oligonucleotides), carbohydrates, glycoproteins, monoclonal antibodies (mAbs) and their fragments, peptides, proteins, or other small molecules adsorbed onto the liposomal surface [22,24]. Figure 3 shows the surface modification of liposomes for active targeting. Figure 4 shows the distinction between passive and active targeting. Table 3 shows some examples of liposomes and their ligands used for active targeting.

### 1.3. Functionalized Liposomes

**Figure 5:**
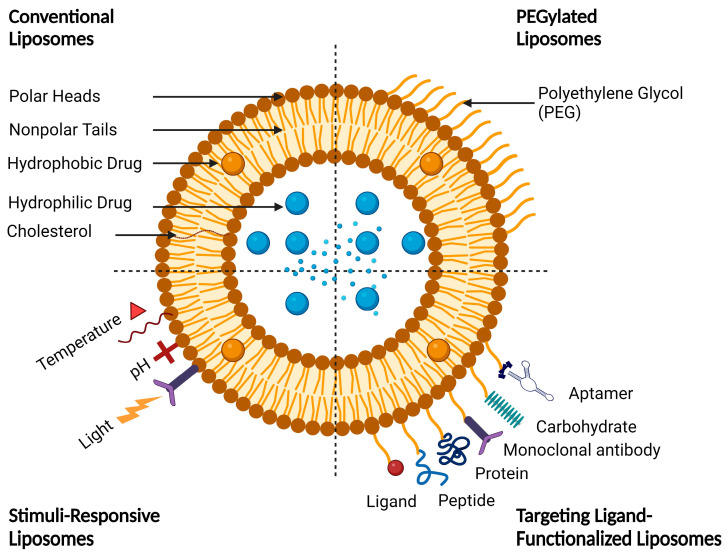
A schematic representation of conventional and functionalized liposomes.

Functionalized liposomes include long-circulating PEGylated liposomes, targeting ligand-functionalized liposomes, and stimuli-responsive liposomes (Figure 5).

#### 1.3.1. Long-Circulating PEGylated Liposomes

Effective targeting requires the design of smart drug delivery systems with a long circulatory half-life (i.e., to remain in the bloodstream for longer periods of time), which means that liposomes must evade uptake by RES organs. This unique character can be imparted onto liposomes by coating their surfaces with polymers that suppress opsonization by plasma proteins [38]. These liposomes are commonly known as stealth or long-circulating liposomes, owing to their stealth properties that make them resistant to recognition and degradation by enzymes and immune systems [38,39]. The most commonly used polymer to prevent liposome opsonization is polyethylene glycol (PEG). The process of PEG attachment to liposomes is called PEGylation [39]. Of note, PEG, commercially known as macrogol, is a hydrophilic, biodegradable polymer with a general formula of H(OCH_2_CH_2_)*_n_*OH, where *n* is the number of oxyethylene groups + 1 [40]. There are different grades of PEG available on the global pharmaceutical market, such as PEG-400, PEG-1500, PEG-4000, PEG-6000, and PEG-20000, that differ in their molecular weights. PEGs with molecular weights of 1000 and higher are solid grades and range in their consistencies from pastes to waxy flakes, while PEGs with molecular weights below 1000 are viscous liquid grades [40]. The adsorption of PEGs onto the liposomal surfaces leads to enhanced blood circulation time, reduced RES uptake, increased biodistribution and target accumulation, and enhanced formulation stability [41,42]. However, despite all these advantages, PEG-functionalized liposomes showed some serious drawbacks. For instance, PEG demonstrates potential immunogenicity owing to the activation of complement in response to antibodies [42,43]. In fact, PEG-based delivery systems support the phenomenon of accelerated blood clearance (ABC), owing to the formation of anti-PEG immunoglobulin M (IgM) antibodies by the spleen after initial administration. Following the second administration, the anti-PEG IgM binds to PEG groups on the surface of liposomes, resulting in the activation of the complement system, which subsequently leads to opsonization of the liposomes by C3 fragments and consequently enhances the cellular uptake of liposomes by the Kupffer cells in the liver, which in turn greatly affects the drug’s bioavailability [43]. Even though the ABC phenomenon poses a significant challenge for certain drugs, it does not pose a critical problem for PEGylated liposomes used in cancer therapy, owing to the high lipid content of liposomes encapsulating anticancer cytotoxic agents [43].

#### 1.3.2. Ligand-Functionalized Liposomes

The adsorption of active targeting moieties on the surface of liposomes has played a significant role in enhancing liposomal accumulation in cancer cells since this structural characteristic helps to increase the therapeutic index of the encapsulated drug, maximize on-target effects, and minimize off-target effects. Briefly, active targeting is a surface modification process where active targeting moieties are adsorbed onto the liposomal surfaces, which substantially help to recognize and bind specifically to target cells through ligand–receptor interactions [44]. Actively targeting liposomes are made by grafting moieties such as aptamers, carbohydrates, glycoproteins, mAbs and their fragments, peptides, proteins, and small molecules adsorbed onto the liposomal surfaces (Figure 3). The targeting moiety can be either inserted directly into the lipid membrane or attached specifically to the distal end of the polymer [38,45]. Aptamers are short single-stranded oligonucleotides of deoxyribonucleic acid (DNA) or ribonucleic acid (RNA) with typically 20–100 base pairs. They have been widely studied as targeting agents to deliver various drugs and photosensitizers with high specificity to cell receptors and binding affinity at the nanomolar level [46]. Aptamers can be easily synthesized chemically and modified for conjugation. Different aptamers recognize different molecular targets. Cell-type-specific aptamers can be designed and synthesized by the exponential enrichment (SELEX) method, which can generate cell-type-specific aptamers with a high target affinity [47]. Transferrin is a blood plasma glycoprotein that plays a key role in iron metabolism by binding iron and transporting it into cells via TFR-mediated endocytosis [48]. Tumor cells often require excess amounts of iron for fast proliferation; thus, transferrin receptors on tumor cells are overexpressed in order to increase the uptake of iron from the plasma [48,49]. Folate (folic acid) is an essential vitamin for DNA synthesis and cell division. Folate and its derivatives have a high affinity for FRs and are internalized by FR-mediated endocytosis [50]. The receptor–ligand interaction affects the rate of cellular internalization, which in turn influences the accumulation of liposomal drugs in the tumor cells. Folate has the advantages of fast internalization and intracellular recycling rate, which subsequently fastens the delivery of drugs and photosensitizers to tumor cells [50,51]. Antibodies can recognize tumor cells by binding to specific antigens overexpressed by tumor cells with high affinity. Antibody targeting has been extensively studied for the delivery of drugs and photosensitizers to tumor cells; however, the therapeutic uses of mAbs are limited owing to antibody recognition and immunologic response [52]. Antibody fragments, such as single-chain variable fragments (scFv) and antigen-binding fragments (Fab), have been developed to modulate the overexpression of specific membrane antigens. Antibody fragments are smaller in size than mAbs, which allows them to efficiently reach their targets [52]. Although peptides have low molecular weights and a lower immunogenicity than antibodies, which lead to improved tumor penetration, they have a lower binding affinity. Peptides can be easily synthesized chemically and modified to obtain the desired physicochemical and biopharmaceutical properties. However, the presence of PEG polymers on the liposome surface may prevent peptides from interacting with cells. Therefore, peptides should be projected away from the liposome surface to avoid the shielding of the PEG polymer [53]. To conclude, the proper selection of targeting ligands is necessary to achieve high binding affinity and specificity. A recent trend in liposome surface functionalization includes the decoration of the liposomal surface with two ligands (i.e., dual-targeting). Dual-targeted liposomes offer numerous advantages, such as targeting multiple receptors, delivering more than one drug to target sites, enabling the encapsulated drugs to exert enhanced therapeutic effects, and reducing normal tissue toxicity and damage [54,55]. Two strategies are commonly used for dual-targeting based on different ligand combinations. The first strategy is based on the combination of two targeting ligands that leads to improved tumor selectivity and the cellular uptake of liposomes by specific tumor cells. The second strategy is based on the combination of targeting ligands with cell-penetrating peptides (CPPs) to achieve selective cellular uptake and enhanced anticancer efficacy toward specific tumor cells [55,56]. A dual-ligand combination involves the use of two ligands to target different receptors, which can be expressed only on one cell or on different cells. These ligands can be combined into one molecule or even placed separately on the surface of liposomes [56]. Common examples of dual-ligand combinations are those in which the second ligand is combined with either RGD, HA, or transferrin, since those ligands are overexpressed on different tumor cells [56]. In general, dual-ligand combinations can be classified into three different classes based on the types of target cells and the target sites of action. In the first class, two ligands are used to target one cell type, which is overexpressed on two receptors. The second class involves the use of two ligands to target two cell types, while the third class combines cell membrane targeting with intracellular organelle targeting (such as nuclear targeting or mitochondrial targeting) [55,56]. In fact, surface-modified liposomes with one or more targeting ligands exhibit improved cellular uptake compared with conventional liposomes due to receptor-mediated endocytosis. However, the cellular uptake efficiency of liposomes by tumor cells is still limited since the receptors on the surface of tumor cells dynamically change with tumor progression, leading to the saturation of receptor–ligand binding [56,57]. The discovery of CPPs helped to overcome these barriers, especially the cell membrane barrier, and subsequently enhanced cellular uptake [57,58]. However, in vivo applications of surface-functionalized liposomes with CCPs are limited since the non-specificity of CPPs may penetrate normal cells and lead to systemic toxicity [59,60]. Furthermore, the surface-functionalized liposomes with CCPs may be easily recognized by the RES owing to the high density of surface positive charge [61]. A new strategy based on the combination of one ligand with one or more stimuli to finely control the liposomes in a specific tumor site will be discussed in this review.

#### 1.3.3. Stimuli-Responsive Liposomes

Liposomes can respond to different internal and external stimuli and thus trigger the release of encapsulated drugs in a controlled manner to specifically target cancer cells. Internal stimuli include enzymes (such as cathepsin B enzyme, a lysosomal protease of the papain family, which is overexpressed in several pathological conditions and malignancies, including the brain, breast, colon, lung, and prostate cancers), pH (such as DOPE that adopts an inverted hexagonal phase II (H_II_ phase) at low pH and a bilayer structure (L_α_ phase) at neutral pH to promote the liposomal membrane permeabilization), redox (e.g., disulfide-containing liposomes that can be easily broken down by reducing glutathione at the tumor site, leading to the complete disintegration of the liposomal membrane), and temperature (i.e., temperature can act either as an external stimulus when heat source is applied from outside, or can be internal when cancer pathological lesions have a naturally elevated temperature) [62,63], while external stimuli include light, electrical fields, magnetic fields, and ultrasound waves [62,63]. Light, in the UV-visible-IR region, is a very promising tool for biological and medical applications due to its non-invasive nature, high spatial resolution and temporal control, tuneability over a wide range of wavelengths, convenience and ease of application, and robustness [64]. In comparison with other stimuli, light provides an unparalleled spatiotemporal modulation of molecular processes [65], making it highly suitable for clinical and therapeutic applications [64,65]. Table 4 summarizes the advantages and limitations of different types of stimuli. Light-responsive liposomes have been recently introduced as smart, intelligent drug targeting delivery systems to target drugs for specific sites with high spatial and temporal control over drug release [66]. These systems utilize nonionizing radiation and are mainly composed of biocompatible, biodegradable materials that can be straightforwardly tailored to the target sites for clinical and therapeutic applications [66]. Although most light-responsive liposomes respond to UV irradiation that has a poor tissue penetration and high phototoxicity, optical technologies like laparoscopic tools are now commonly used for reaching deeper located tissues [64,65]. On the other hand, NIR is safer for use, causes less cell damage, and has good tissue penetration. However, the lower energy of NIR may not be efficient enough to induce the desired drug release response from liposomes [67,68].

In this review, the up-to-date targeting strategies and mechanisms of light-triggered drug release from liposomes and NIR-responsive nanocarriers are discussed in light of surface functionalization and target structures. Moreover, we highlight recent key advances in the design and application of light-responsive liposomes and dual-targeted stimuli-responsive liposomes. Lastly, we outline the current challenges and future perspectives for the deployment of light-responsive liposomes in targeted drug delivery and therapy. Our main goal is to provide a step towards developing the next generation of light-responsive liposomes and dual-targeted stimuli-responsive liposomes.

## 2. Mechanisms of Light-Triggered Drug Release from Liposomes

**Figure 6:**
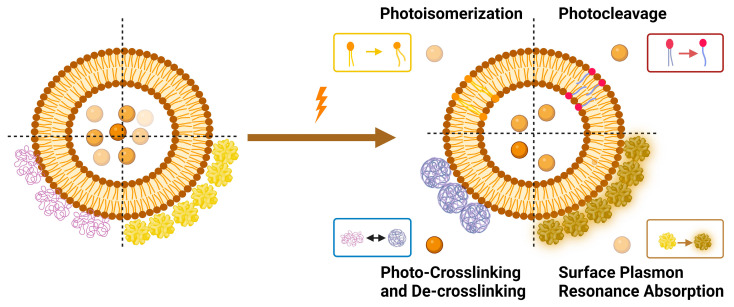
Light-triggered mechanisms used in triggering drug release from liposomes.

Light-triggered mechanisms that can be exploited to release encapsulated drugs from liposomes are photoisomerization, photocleavage (photo-oxidation), surface plasmon resonance absorption (photothermal activation), photochemical hydrophobicity change (photochemical activation), and photo-crosslinking and de-crosslinking (Figure 6).

### 2.1. Photoisomerization

Photoisomerization is a photo-induced isomerization process from one isomeric form to another (i.e., *cis (Z)-* to *trans (E)-*isomer). It is worth mentioning that *trans (E)-*isomers are more stable and lower in energy than *cis (Z)-*isomers due to no electrical repulsion since the two larger groups are as far away as possible from each other, while in the case of *cis (Z)*-isomers, the two larger groups bump into one another, resulting in an electrical repulsion [79]. When photo-responsive molecules are irradiated with UV light, they undergo conformational changes from *trans*- to *cis*-isomers. These conformational changes make the structural integrity of liposomes more permeable, owing to the steric hindrance as well as the increased polarity of *cis*-isomers [79]. The transition from *trans*- to *cis*-isomers can be triggered by UV light irradiation at wavelengths ranging from 320 to 350 nm, and the reverse transition can be triggered by visible light irradiation (400–450 nm) or by heat.

**Figure 7:**
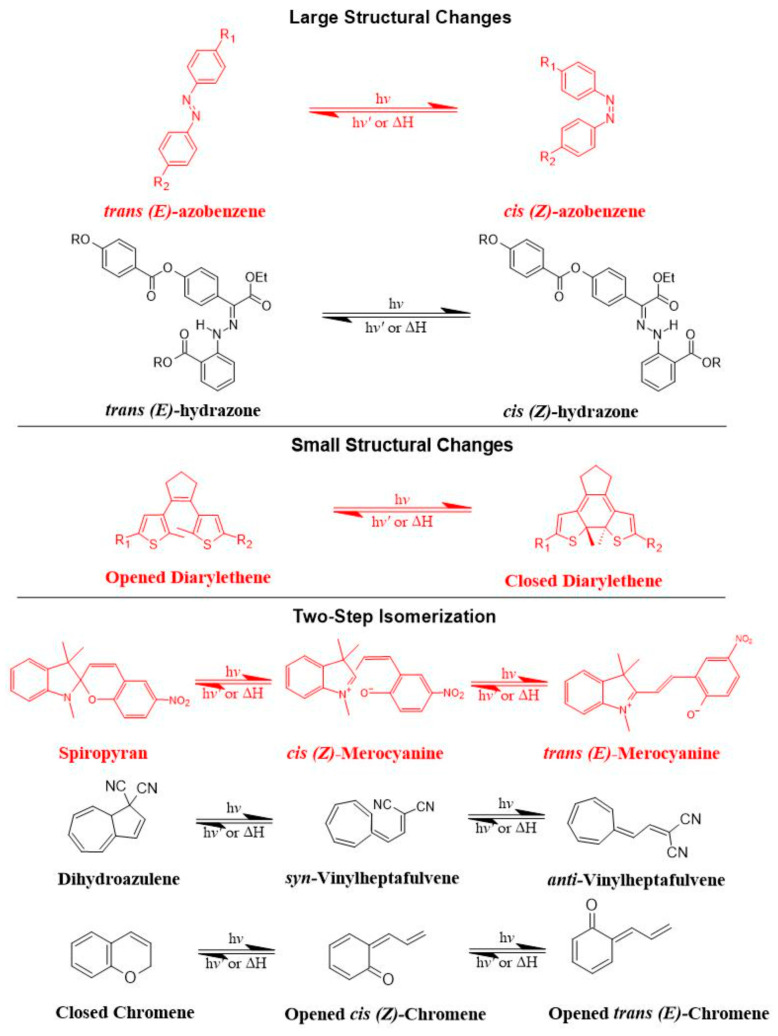
The most common photoswitches and their photoisomerization reactions.

Azobenzene, spiropyran, and diarylethene are the most commonly used photoswitches in photoisomerization-based drug release [80,81]. Azobenzene undergoes a UV-light-induced double-bond isomerization to its metastable *Z*-isomer, which is characterized by being shorter in length, bent, twisted, and more hydrophilic than the *E*-isomer. Spiropyran (carrying a neutral charge) undergoes a UV-light-induced ring-opening reaction to its zwitterionic metastable form, which is commonly known as merocyanine and is characterized by being more hydrophilic. While diarylethene undergoes a UV light (6π) electrocyclization and ring-closing reaction to its thermally stable isomer, which is characterized by being conjugated and rigid in structure, the ring-closed isomer can be reopened again using visible light (Figure 7) [80,81].

There are preferred application areas for each photoswitch. For example, azobenzenes are superior photoswitches when large structural and geometrical changes are required [82]. Complementary to azobenzenes, diarylethenes show small structural and geometrical changes but large electronic changes upon photochemical interconversion between the ring-opened and -closed structures [83]. Spiropyrans offer unique properties with respect to ring-opening and -closing isomerization, owing to their molecular dipole moments, which increase during photoconversion processes from ring-closed to -opened structures (Figure 7) [84].

The mechanism of *trans-cis* photoisomerization has been used to induce drug release from light-responsive liposomes. As an interesting example of the photo-response mechanism of the azobenzene photoswitch in liposomes, Li et al. [85] developed a novel liposomal curcumin formulation with photoswitching properties, owing to the presence of 4-butylazobenzene-4-hexyloxy-trimethyl-ammoniumtrifluoro-acetate (BHA) as a photo-responsive reversible switch. The azo-group of BHA was capable of undergoing a reversible *trans-cis* isomerization under UV and visible light irradiation. BHA-curcumin-liposomes, abbreviated BHA-cur-lipo, were prepared using the thin-film hydration method along with the SC-CO_2_ technique. The percent encapsulation efficiency (EE%) of curcumin in BHA-cur-lip was ~88%. Curcumin was released from BHA-cur-lipo under UV light irradiation; ~90% curcumin was released within 6 h. BHA embedded in the liposomal bilayer was able to isomerize under UV light irradiation, and the isomerization process was capable of being repeated multiple times. The isomerization of BHA in the liposomal bilayer could be used as a switch for precisely controlled, on-demand drug release.

As an interesting example of the photo-response mechanism of spiropyran photoswitch in liposomes, Zhang et al. [86] developed photo-responsive liposomes composed of spiropyran-containing triazole-phosphatidylcholine (SPTPC). SPTPC was synthesized through a copper-catalyzed azide alkyne cyclo (CuAAC)-addition reaction. In an aqueous solution, SPTPCs self-assembled into vesicles due to the presence of phosphatidylcholine (PC), and then a spontaneous isomerization of spiropyran-to-merocyanine (SP-to-MC) occurred, resulting in the co-occurrence of liposomes and fibers. The switching from spiropyran (SP) to merocyanine (MC) isomeric form induced a reversible transition between these molecular structures. Additionally, the authors studied the self-assembly properties of SPTPCs and photoinduced liposome–fiber assembly-transition and concluded that (1) the presence of MC allowed for additional intermembrane interaction during self-assembly, and (2) the driving force for the assembly-transition was the MC-stacking effect. Exposure to UV light at 365 nm induces switching from SP to MC isomeric form, where the planar structure and confinement of MC lead to enhanced MC-stacking. The MC-stacking effect had some advantages and drawbacks, such as disrupting the hydrophobic phase in the lipid bilayer and permitting the liposome-to-fiber transition; otherwise, the MC-stacking blocked the switching of MC to SP and caused an incomplete isomerization recovery from MC to SP during fiber-to-liposome recovery. Therefore, a fatigue of SP was observed during the liposome-to-fiber transition cycle. To suppress the MC-stacking effect and minimize the intermolecular interaction, a photo-inert triazole-phosphatidylcholine (TPC) was subsequently added to make TPC/SPTPC-liposomes, which showed better recovery kinetics. The active photoadaptation behavior of TPC/SPTPC-liposomes confirmed the disturbance of the lipid bilayer through the formation of MCTPC-enriched phases in the lipid bilayer. To conclude, the reversible liposome-to-fiber assembly transition of SPTPC is a promising and potential candidate for adaptive assembly systems.

As an interesting example of the photo-response mechanism of diarylethene photoswitch in liposomes, Liu et al. [87] synthesized a novel amphiphilic photoswitchable fluorescent probe of liposomes, namely, PEGylated perylenemonoimide-dithienylethene, abbreviated PEG-PMI-DTE, that exhibited excellent photochromic reversibility, fluorescence switching, and fatigue resistance under UV and visible light irradiation. The fine nanostructures of liposomes (MLVs, LUVs, and SUVs) were able to be observed directly under a super-resolution optical microscope with the use of amphiphilic photoswitchable fluorophore as a staining agent, with an optical resolution of 30 nm. This research offers a new type of optical probe and an optical approach to investigating nanostructures using photoswitchable fluorescent probes in super-resolution imaging.

### 2.2. Photocleavage (Photo-Oxidation)

Photocleavage is a photo-induced bond cleavage through photosensitized oxidation that can be achieved when the photosensitizer and oxygen are proximate to the oxidizable lipid in liposomes, which are characterized by having a lipid segment sensitive to singlet oxygen (^1^O_2_) produced by the photosensitizer [88]. The photocleavage release mechanism from liposomes occurs mainly through lipid photo-oxidation [89], which leads to membrane destabilization, disruption, and subsequently drug release [89]. Briefly, when liposomes are irritated with light, the photosensitizer will absorb photos, leading to an excited triplet state. This generates reactive oxygen species (ROS) that can be either in the form of radicals (hydroxyl (HO^•^) and superoxide (O_2_^•^)) or non-radicals (^1^O_2_). Singlet oxygen (^1^O_2_) is a highly reactive oxidant with low stability and a short half-life. It can oxidize different cellular constituents, such as nucleic acids, lipids, and proteins [88,89,90]. Its tendency to induce toxicity can be precisely controlled.

The mechanism of photocleavage was explored through photodynamic therapy (PDT). PDT is a light-based cancer therapy that uses light to activate photosensitizers, leading to the generation of ROS, or ^1^O_2_, which are highly reactive oxidants that can mediate damage to tumor cells or tissues. The effectiveness of PDT depends on several factors [91,92], such as (1) the type of photosensitizer, (2) the intensity of light, (3) the route of administration, (4) tumor type, size, and location, and (5) the concentration of dissolved cytoplasmic oxygen. The ideal photosensitizer should be [91,93,94] (1) safe, effective, and non-toxic; (2) a water-soluble compound; (3) pharmacologically inactive in the absence of a light source; (4) highly specific and selective; (5) have an absorption spectrum preferably between 650 nm and 800 nm; and (6) be rapidly metabolized to metabolites and discharged from the human body. Photosensitizers are categorized into two main classes: porphyrin photosensitizers and non-porphyrin photosensitizers [94]. Three generations of porphyrin photosensitizers exist. First-generation porphyrin photosensitizers include hemaporphyrins, which have several drawbacks that limit their therapeutic use, such as (1) chemical instability issues; (2) poor tissue penetration; (3) activation with light below 650 nm; (4) skin hypersensitivity reactions; (5) long half-life; and (6) low elimination rates [94]. Second-generation porphyrin photosensitizers include metalloporphyrins, porphycenes, purpurins, chlorins, and protoporphyrins [94]. Second-generation porphyrin photosensitizers have been approved by the FDA and EMA for the treatment of cancer. For example, 5-aminolevulinic acid (ALA) and methyl aminolevulinate (MAL, Metvix^®^, Galderma, Lausanne, Switzerland) are precursors of protoporphyrin IX, which absorbs at 630 nm. They are approved by the FDA for the treatment of prostate, bladder, and colon cancers [20]. Meta-tetrahydroxy-phenyl chlorine (m-THPC, Temoporfin, Foscan^®^, Biolitec Pharma, Vienna, Austria) absorbs at ~652 nm and is approved by the EMA for the treatment of biliary and pancreatic cancers [21]. Verteporfin (Visudyne^®^, Novartis, Basel, Switzerland), a benzo-porphyrin derivative, absorbs at 690 nm and is approved by the FDA for the treatment of gastric cancer [20]. Coupling the second-generation porphyrin photosensitizers with biologically targeting molecules, such as carbohydrates, peptides, or antibodies, resulted in the third-generation porphyrin photosensitizers, which displayed high selectivity and specificity with minimal adverse effects [94]. Non-porphyrin photosensitizers include psoralens, anthracyclines, chalcogenopyrylium dyes, cyanines, and phenothiazinium dyes [94]. Although all of the above advantages of PDT photosensitizers in the treatment of cancer exist, they are still suffering from serious drawbacks and limitations, such as poor biodistribution and cellular uptake of hydrophobic photosensitizers, difficulty in applying PDT to deeper tumor tissues, and low sensitivity and selectivity towards some cancer cells [91,92,93,94]. Therefore, PDT is only effective and suitable for treating superficial skin tumors.

Since the use of photosensitizers often causes serious skin hypersensitivity reactions, the encapsulation of photosensitizers into nanocarrier systems, such as liposomes, will overcome these problems. Of note, Sun et al. [95] developed anticancer liposomal chemophototherapy (CPT) using a bilayer-loaded photosensitizer and the anti-cancer drug cabazitaxel (CTX). Cabazitaxel-loaded porphyrin–phospholipid liposomes, abbreviated CTX-PoP-Lip, were prepared using the hot ethanol injection method in order to encapsulate the hydrophobic CTX within the lipid bilayers. Cholesterol and PEG-lipid were added to enhance liposomal stability and permeation. The EE% of CTX in CTX-PoP-Lip was ~60%, and the percentage of loading capacity (LC%) was ~2%. Morphologically, CTX-PoP-Lip showed spherical, unilamellar vesicles with a diameter size of ~100 nm. CTX-PoP-Lip showed an optical absorption peak similar to PoP-Lip without CTX, with a characteristic PoP peak apparent at 420 nm (for the PoP Soret band) and 675 nm (for the PoP Q-band). Upon excitation at 675 nm, a fluorescence peak was observed for both CTX-PoP-Lip and PoP-Lip. Without PoP, CTX-Lip had no fluorescence. Over 3 months of storage under 4 °C, CTX-PoP-Lip displayed good colloidal stability in terms of particle size, polydispersity, and zeta potential. Moreover, CTX showed good photochemical stability under laser irradiation. Remarkably, the combination of CTX-PoP-Lip with laser treatment showed a positive tumor inhibition therapeutic effect in comparison with PDT alone or chemotherapy alone.

Lipid–porphyrin conjugates are novel, promising carriers for drug delivery with multifunctional and light-triggered release properties. These compounds are able to self-assemble into liposome-like structures to form porphysomes that consist of porphyrin–lipid conjugates generated by acylation reactions between phospholipids and chlorophyll-derived porphyrin analogues [96]. As a result of this supramolecular self-assembly, the porphysome bilayer contains an extremely high porphyrin packing density, resulting in extreme self-quenching of Pyro fluorescence [96]. Upon NIR irradiation, the energy absorbed is released primarily as heat, generating temperatures in magnitudes comparable to those of inorganic nanoparticles. This large heat generation makes porphysomes a very promising photothermal sensitizer [97]. Moreover, the structure-dependent self-quenching properties of porphysomes enable fluorescence imaging upon the disruption of the porphysome bilayer. As porphysomes are captured by cells via endocytosis, the structure of porphysomes is disrupted, and fluorescence is greatly increased. This activatable fluorescence indicates that the porphysomes have reached the desired target site [96,97]. On the other hand, the porphyrin rings offer chelating sites for metal atoms, thus making porphysomes potential candidates for the development of multimodal imaging contrast agents. Alongside these photosensing properties, porphysomes possess liposome-like characteristics with improved photophysical properties [98]. For instance, porphysomes can be actively targeted at tumor cells by modifying their surfaces through any of the active targeting strategies used by conventional liposomes, as previously described [99].

**Figure 8:**
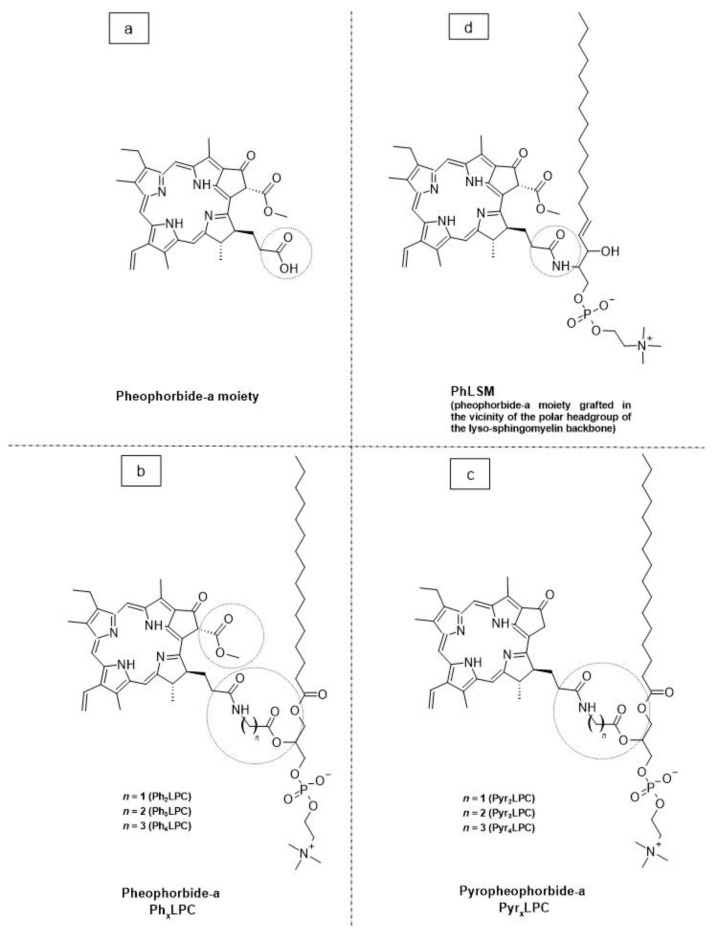
The chemical structures of (**a**) pheophorbide-a; (**b**) pheophobide-a PhxLPC; (**c**) pyropheophorbide-a (Pyr_X_LPC); and (**d**) PhLSM.

As a promising example of these self-assembled structures, Cressey et al. [100] developed novel liposome-like assemblies composed of newly synthesized phospholipid–porphyrin conjugates bearing either pheophorbide-a (Pheo-a) or pyropheophorbide-a (Pyro-a) photosensitizers. These conjugates presented different alkyl chain lengths in the *sn-2* position and were linked to either pheophorbide-a (Ph_x_LPC) or pyropheophorbide-a (Pyr_x_LPC) via amide coupling (Figure 8). These liposome-like structures were composed of lipid–porphyrin conjugates made of cholesterol, a phospholipid–porphyrin conjugate, and DSPE-PEG-2000 in a ratio of 47.5:47.5:5 mol% and were prepared using the thin-film hydration method, followed by the extrusion of the vesicles. Interestingly, all phospholipid–porphyrin conjugate assemblies showed similar absorbance and fluorescence, with higher fluorescence quenching for Pyro-a conjugates. Formulations containing the phospholipid–porphyrin conjugate with the longest linker displayed higher stability than those with a shorter one. This could be due to the deeper embedment of the porphyrin core inside the lipid matrix. Based on these results, the authors selected Pyr_3_LPC and Ph_3_LPC liposomes as promising candidates for in vitro studies. Another very promising example is Massiot et al. [101], who designed photo-triggerable liposomes based on the lipid–porphyrin conjugate and cholesterol combination. First, they synthesized a new lipid–porphyrin conjugate, termed PhLSM, by coupling pheophorbide-a (Pheo-a) with egg lyso-sphingomyelin (Figure 8). The pure PhLSMs were able to self-assemble into liposome-like structures, but they were highly unstable due to the mismatch between the length of the alkyl chain in the *sn*-1 position and the adjacent porphyrin. Stable PhLSM lipid bilayers were obtained by mixing PhLSMs with cholesterol, which is known to exhibit a complementary geometrical packing parameter. Based on these observations, the authors prepared stable liposomes encapsulating a hydrophilic fluorescence probe in the aqueous core. The prepared liposomes showed light-triggered cargo release in an ON/OFF fashion, which was attributed to their photothermal conversion. In addition to the light-triggered cargo release property and phototoxic photothermal effect, the prepared liposomes showed a markedly high photothermal conversion efficiency and photostability.

### 2.3. Surface Plasmon Resonance Absorption (Photothermal Activation)

**Figure 9:**
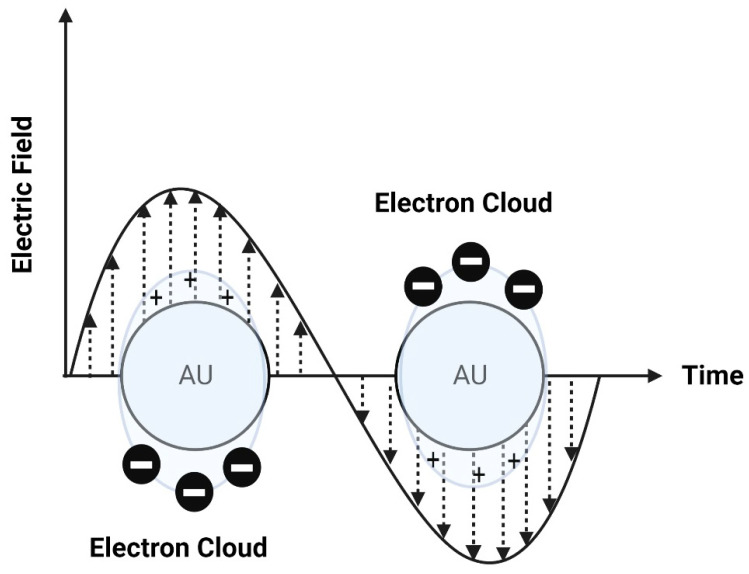
A schematic diagram of surface plasmon resonance (SPR).

Photothermal approaches involve the conversion of light into heat to induce liposomal membrane permeabilization. Some metals exhibit unique optical properties, such as dielectric function, reflectivity, and electron energy loss function, when present in the form of nanostructures as nanoparticles or entrapped inside the nanocarrier systems as nanoparticle-loaded liposomes [102]. Metallic nanostructures are highly attractive multifunctional nanoplatforms, owing to their unique size- and shape-dependent properties [102]. One of the most interesting characteristics of metallic nanostructures is their optical properties, which are strongly dependent on particle size and shape [102]. For example, bulk gold metals look yellowish in reflected light, but thin gold films look blue in transmission. This characteristic blue color gradually changes to orange through several tones of purple and red as a result of reducing particle size to ~3 nm. These changes are likely to account for the surface plasmon resonance (SPR) [102], which is defined as the frequency or wavelength at which conduction electrons oscillate with regard to the alternating external electric field (Figure 9). The optical properties of metallic nanostructures are controlled by the collective oscillation of conduction electrons, resulting from the interaction with the electric field of the incident light, owing to the presence of free conduction electrons [103]. The electric field of the incoming radiation creates a strong dipole electric field inside the metallic nanostructures. A restoring force in the metallic nanostructures attempts to compensate for this difference, resulting in a unique resonant wavelength [103].

The SPR frequency and intensity of metallic nanostructures are dependent on the electron charge density, which is primarily affected by several factors, such as size, shape, structure, composition, and the dielectric constant of the surrounding environment.

Interestingly, Rubio-Camacho et al. [104] synthesized stable gold nanoparticles on the surface of DPPC thermosensitive liposomes, termed AuNPs@DPPC, resulting in the formation of nanohybrids with an on-demand plasmon mode in the visible/NIR region and with good photothermal conversion efficiency. The AuNPs@DPPC nanohybrids retained the physical properties of DPPC thermosensitive liposomes without altering either the liposome fluidity or the hydration degree of the lipid bilayer. The AuNPs@DPPC nanohybrids showed good light-to-heat conversion properties upon irradiation in the NIR region. These nanohybrids represented highly attractive and promising candidates in light-mediated therapies, such as NIR-light-controlled drug delivery. As an interesting example of gold nanoparticle–drug conjugates in liposomes, Li et al. [105] synthesized vincristine sulfate-conjugated gold nanoparticles incorporated into liposomes as a promising light-responsive hybrid nanocarrier system with enhanced antitumor efficiency. Gold nanoparticles were synthesized by reducing tetrachloroaurate, using trisodium citrate as the reducing agent. The amount of trisodium citrate used in the synthesis of gold nanoparticles was 1% to prepare uniform size-controlled nanoparticles. The resulting gold nanoparticles had a particle size of 17 nm. The conjugation of gold nanoparticles with vincristine sulfate was achieved via ionic bonding, since vincristine sulfate is positively charged while the citrate-capped gold nanoparticles are negatively charged. The highest EE% was achieved when the vincristine–gold nanoparticle molar ratio was 6:100. The conjugates were incorporated into liposomes using film dispersion to yield nanoparticles of 113.4 nm with UV light-responsive, controlled release properties. Interestingly, UV irradiation also considerably increased intracellular drug release, cytotoxicity, and apoptosis in HeLa cells. In vivo studies in tumor-bearing nude mice showed that the therapeutic efficacy of vincristine was enhanced after exposure to UV light, with a relatively high tumor inhibition rate and low toxicity. The accumulation of the drug selectively at the tumor site (by the EPR effect of liposomes), together with light-responsive controlled release, represented an important step forward in tumor-targeting drug delivery.

### 2.4. Photochemical Hydrophobicity Change (Photochemical Activation)

Amphiphilic block copolymers have a relatively high potential to produce nanostructures (either micelles or vesicles) via self-assembly in suitable solvent systems [103]. Polymeric micelles are thermodynamically stable when the concentration of polymers is above the critical micelle concertation (CMC) value. If the concentration of polymers is below the CMC value, micelles will disintegrate, dissolve, and release their payloads. Thus, in such cases, polymeric micelles are thermodynamically instable [103]. Therefore, various techniques were developed and applied to improve the thermodynamic stability of polymeric micelles. Foremost among these techniques is a photochemical activation method based on changing the hydrophobicity of molecules [103,106]. Briefly, this mechanism depends on increasing the CMC value and dissolving the micelles by converting the amphiphilic polymers to more hydrophilic forms, thus providing a controlled drug release [103,106]. Interestingly, light-responsive chromophores, such as azobenzene, spiropyran, diarylethene, and their derivatives, can be incorporated inside the micellar cavity, where NIR can be used to induce chemical transformation to a more hydrophilic form [106]. Most light-responsive chromophores can absorb UV light; however, NIR is more suitable for biomedical applications owing to its capability to penetrate deeply into tissues (up to 10 cm) with a low potential for tissue damage [106]. Self-assembled polymeric micelles are used as amphiphilic particulate emulsifiers for controllable Pickering emulsions. Pickering emulsions have been developed unprecedently in drug delivery. However, engineering tunable Pickering emulsions with the capability of responding to light still remains very challenging. Interestingly, Zhao et al. [107] designed a photo-controllable nanocarrier system to control the amphiphilicity of Pickering emulsifiers using a *β*-cyclodextrin-grafted alginate polymer and an azobenzene derivative. Briefly, a biocompatible alginate polymer grafted with *β*-CD (via the Ugi reaction), abbreviated Ugi-Alg-CD, was first synthesized and used as an amphiphilic macromolecule surfactant host. Then, azobenzene coupled with polyethylene glycol (Azo-PEG) was prepared and used as a guest molecule. By coupling Ugi-Alg-CD with Azo-PEG, a stable Pickering emulsion was successfully fabricated. The photoisomerization of a host–guest complex between *β-*cyclodextrin and an azobenzene derivative was customized to regulate the polarity of the microenvironment. Interestingly, the photoactivatable emulsifier based on supramolecular self-assemblies was able to undergo destabilization of O/W emulsions by changing the amphiphilic balance of host–guest assemblies at the O/W interface under UV light irradiation, resulting in phase separation. The analysis of the microstructures of self-assemblies at the O/W interface during the demulsification process indicated that the reversible light-triggered *trans-cis* isomerization of Azo-PEG likely resulted in the regulation of the hydrophilic-hydrophobic balance of supra-amphiphilic polymer emulsifiers. This photochemical strategy opened the door to developing novel photo-responsive nanocarrier systems for various biomedical applications. However, to the best of our knowledge, this mechanism has not been reported in conventional liposomes since it requires the presence of amphiphilic block copolymers in the liposome structure.

Polymersomes are biomimetic cell membrane-like bilayer vesicles that are self-assembled stepwise from amphiphilic block copolymers. They are analogous to liposomes but with outstanding properties, such as a higher chemical stability towards oxidation and hydrolysis reactions and a greater resistance to mechanical deformation processes within the human body like bending and stretching (i.e., resistance to high shear rates of blood circulation and deformations during blood flow through microvessels) or cellular processes (e.g., division and fusion) [108]. Moreover, other properties, such as composition, size, shape, and surface chemistry, resulted in increased EE% and LC% (i.e., polymersomes have a lower membrane fluidity and higher viscosity due to the presence of amphiphilic block copolymers), which contribute to the low permeability of encapsulated drugs from the inner core of polymersomes to the outer site [108,109]. Polymersomes can disassemble in response to light to control the release of encapsulated drugs that may also respond to light. Thus, polymersomes can provide spatiotemporal control of drug release. Interestingly, Yamamoto et al. [110] studied the structure–function relationships and photo-release characteristics of different types of photo-responsive polymersomes composed of amphiphilic di-block copolymers. The building blocks of these photo-responsive polymersomes were hydrophobic polymers and poly(ethylene glycol) with photocleavable 2-nitrobenzyl compounds bearing alkyne and maleimide moieties. Interestingly, all polymersomes preserved their hollow structures even after light irradiation. Additionally, polymersomes with a 2-nitrosobenzyl photolysis residue within the hydrophobic shells showed photo-induced drug release after complete photolysis. The authors concluded that the drug release was controlled by photo-induced permeability changes of the hydrophobic shells rather than the decomposition of their molecular structures.

### 2.5. Photo-Crosslinking and De-Crosslinking

The mechanism of photo-crosslinking-induced drug release occurs through the polymerization of unsaturated bonds located in the hydrophobic domain of the lipid bilayer. When photo-responsive polymerizable moieties are irradiated with light at a specific wavelength, the crosslinking reaction between them causes the shrinkage of the lipid bilayer in the surrounding domain where the photosensitizers are present. This causes bilayer disruption by altering lipid packing; as a result, conformational changes occur, leading to increased membrane permeability and drug release rates [103,106]. The mechanism of photo-crosslinking was first reported in liposomes by Regen et al. [111]. Liposomes were prepared with a photo-triggerable lipid containing two methacrylated phosphatidylcholine derivatives. The resulting liposomes were more stable than the non-crosslinked type and displayed prolonged blood circulation and enhanced tumor accumulation and retention. More interestingly, Nakamura et al. [112] described the transportation of DNA into liposomes using ultrafast photo-crosslinking. The cohesion of the DNA adsorbed onto the liposomal surface induced transformations in the liposomal structure and allowed photo-triggered, sequence-specific DNA transportation into liposomes. This technique was a useful tool for the specific delivery of nucleic acid drugs.

Reversible photo-decrosslinking is a promising emerging alternative to optimize target-specific drug binding. Photo-decrosslinking was first reported in 2009 by He et al. [113], who formulated a nanogel made with a di-block copolymer (PEO-*b*-P(MEOMA-co-CMA)) composed of polyethylene oxide (PEO) and a coumarin-containing poly(2-(2-methoxyethoxy)ethyl methacrylate), P(MEOMA-co-CMA). Crosslinking was achieved by using UV light at 310 nm, while de-crosslinking was achieved by irradiating at 260 nm. Recently, Lu et al. [114] developed a photo-responsive microgel that can be reversibly photo-crosslinked and de-crosslinked using UV light of two different wavelengths. This microgel was prepared through the precipitation copolymerization of 2-(2-methoxyethoxy)ethyl methacrylate (MEO_2_MA), methacrylic acid (MAA), and 7-(2-methacryloyloxyethoxy)-4-methylcoumarin (CMA). The effective crosslinker CMA can be photo-crosslinked through irradiation with UV light at 365 nm and photo-decrosslinked through irradiation with UV light at 254 nm. To understand the photoswitching mechanism, the volume-phase transition temperature (VPTT) was monitored during transitions. The authors concluded that there was a significant change in VPTT that led to a uniform distribution of CMA within the microgel interior. The photo-induced swelling behavior of the microgel was employed to control the release of the anticancer drug doxorubicin. This research study opened the door to developing new hybrid systems of liposome-in-gel as promising carriers for cancer therapy.

## 3. Mechanisms of NIR Light-Triggered Drug Release

**Figure 10:**
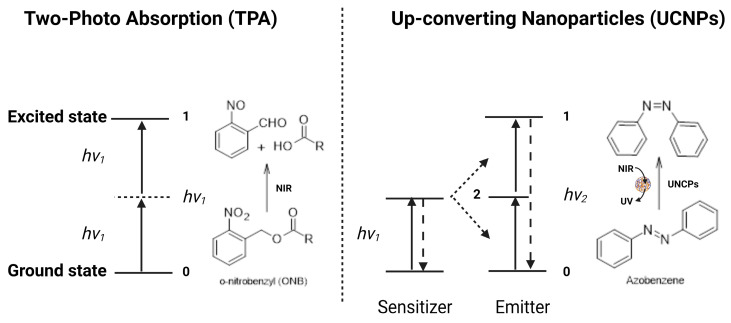
A schematic representation of the energy transfers processes of two-photon absorption and NIR-mediated up-conversion.

Light-triggered drug release from liposomes is mainly dependent on the penetration depth of the selected light source, the photophysical properties of the incorporated photo-responsive molecule, as well as the chemical composition and surface properties of the liposomal nanocarrier [115]. Several radiations have been used to trigger drug release, such as UV, visible, and NIR. However, the preferred wavelengths for therapeutic and biomedical applications are found in the NIR region (~700 to 1100 nm), since at these wavelengths, the light penetration depths are more than 1 cm [116]. The main mechanisms of drug release from NIR-responsive liposomes are photothermal effect, two-photon absorption (TPA), and up-converting nanoparticles (UCNPs) (Figure 10).

### 3.1. Photothermal Effect

The photothermal effect encompasses the conversion of light to heat by a photothermal agent loaded inside the liposomal nanocarrier. This heat stimulates the heat-responsive material inside the liposomal nanocarrier and disrupts the liposomal structure, either by disturbing the hydrophilic-lipophilic balance (HLB) or by creating a phase transition that leads to drug release at the target site. For example, Li et al. [117] prepared nanostructured lipid carriers (NLCs) encapsulated in liposomes containing the hydrophilic CXCR4 antagonist plerixafor (AMD3100) and the hydrophobic NIR-photothermal agent IR780. The NIR light stimulated IR780 to produce heat that caused the disruption of the lipid bilayer, resulting in complete nanocarrier disassembly and subsequent drug release. In addition, IR780 induced cytotoxic hyperthermia as a synergistic effect along with chemotherapy. More interestingly, Refaat et al. [118] developed a NIR-activated thermosensitive liposome encapsulated with ultrasmall gold nanorods and a non-ionic surfactant (Brij^®^ 58) for protein delivery. The prepared nanohybrid carrier system showed a significant increase in thermosensitivity due to the thermosensitive property of gold nanorods, which resulted in the rapid release of the encapsulated proteins. Consequently, this system was selected for the encapsulation, on-demand release, and delivery of the thrombolytic agent urokinase-plasminogen activator (uPA). Urokinase light-responsive liposomes exhibited enhanced thrombolytic effect (80.7% lysis of an in vitro halo-clot model in 30 min following NIR irradiation (785 nm, 1.35 W/cm^2^ for 5 min)) compared to free uPA and non-irradiated liposomes (36.3% and 15.5%, respectively). To conclude, the newly engineered, gold nanorod-based NIR light-responsive liposomes represented a promising drug delivery system for on-demand, site-directed, and photothermally stimulated therapeutic protein release.

### 3.2. Two-Photon Absorption (TPA)

Two-photon absorption (TPA) relies on the excitation mechanism induced by two absorbed photons [119]. Briefly, the chromophore is excited from its ground state to its excited state by simultaneously absorbing two photons with equal energy, and then it undergoes a specific photochemical reaction [120,121]. In the case of chromophores with a wide NIR absorption spectrum, such as 2-diazo-1,2-naphthoquinone (DNQ), coumarin, and o-nitrobenzyl (ONB), they can be initiated by NIR light to undergo specific photoreactions, such as rearrangement and photocleavage reactions via the TPA process. Then, the chromophore-functionalized nanocarriers will be destroyed as a result of the changes in molecular structures, leading to drug release (Figure 10). For example, Sun et al. [122] developed NIR-responsive liposomes composed of cholesterol, the NIR-responsive lipid made by incorporating the NIR-light-responsive 6-bromo-7-hydroxy-4-hydroxycoumarin (Bhc) unit into the lipid acyl chain, and POPC. The prepared liposomes were able to encapsulate the hydrophilic molecules in the liposome interior cavity and release their cargos upon NIR irradiation. Drug release from liposomes was controlled by adjusting the percentage of photo-responsive lipid or through irradiation parameters (time and intensity), demonstrating a potential controlled drug release action. This study provides evidence for developing efficient photo-responsive drug and gene delivery systems.

Although TPA is a promising technique for controlled drug delivery, owing to the high spatial and temporal resolution, deep tissue penetration, and low scattering of NIR light, this technique requires a focal pulsed laser with high energy density in order to treat a small infection area. Thus, this method is not suitable for in vivo experiments.

### 3.3. Up-Converting Nanoparticles (UCNPs)

The up-converting nanoparticle (UCNP) process encompasses the conversion of NIR to UV light [123]. Briefly, UCNPs are a process of multi-photon excitation that involves at least two excitation photons, where the absorption of these photons is sequential and not simultaneous. The low-energy NIR can be converted into high-energy UV light by the UCNPs, which would isomerize the azobenzene chromophore from *trans-* to *cis-*isomer (Figure 10) [124]. For more efficient energy transfer, the emission band of UCNPs should overlap the absorption band of the chromophore as much as possible [125]. For example, Xiang et al. [126] prepared UCNPs with an amphiphilic di-block copolymer containing a UV-sensitive inner hydrophobic layer composed of poly(4,5-dimethoxy-2-nitrobenzyl methacrylate) and an outer hydrophilic layer composed of poly(methoxy polyethylene glycol monomethacrylate). When UCNPs were irradiated with NIR light at 908 nm, the amphiphilic di-block copolymer absorbed the UV light and induced a disturbance of the HLB, leading to rapid nanocarrier disassembly and drug release [126]. Once the poly (4,5-dimethoxy-2-nitrobenzyl methacrylate) (PNB) absorbed UV light, the hydrophobic block polymer converted into a hydrophilic block polymer, leading to the dissolution of the di-block copolymer and the release of the drug molecules. Some NIR-responsive carrier systems use a photosensitizer to create a synergistic effect by producing ROS in addition to chemotherapy [127,128].

## 4. Strategies for Light-Targeting Drug Delivery

Regarding strategies for light-targeting drug delivery, three main strategies are generally employed, which are light-targeting through the activation of targeting ligands, light-targeting through particle size reduction, and light-targeting through blood vessel disruption.

### 4.1. Light-Targeting through the Activation of Targeting Ligands

**Figure 11:**
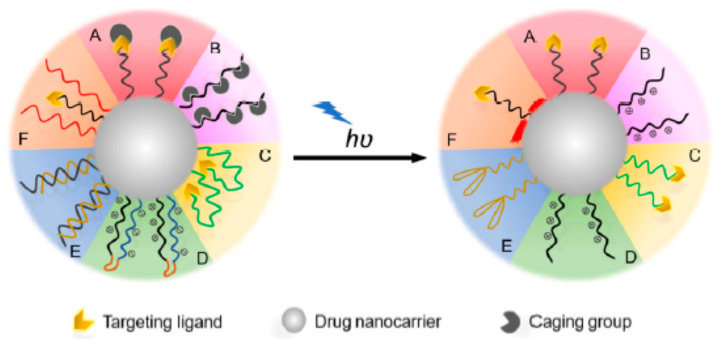
A schematic representation of the light-targeting mechanism through the activation of targeting ligands. **A:** Caging of the ligand binding sites; **B**: caging of the ligand electrical charge; **C**: anchoring of the ligands inside the nanocarrier system; **D**: neutralizing the ligand charge by electrostatic interactions; **E**: shielding of aptamer ligands through the use of complementary oligonucleotides; **F**: shielding of the ligands using thermo-responsive polymers. In all the aforementioned cases, the ligand is either caged or shielded in the nanocarrier system. Upon light irradiation, it is exposed to the surface of the nanocarrier system to allow for active targeting. Figure adapted with permission from [129].

Light-targeting through the activation of targeting ligands enables the active targeting of liposomes through the activation of targeting ligands present on the surface of liposomes, leading to cellular binding following light irradiation. In order to develop a liposomal nanocarrier with light-triggered active targeting, it should (1) temporarily deactivate targeting ligands circulating in the blood stream and (2) expose the targeting ligands following light irradiation at a specific target site [129]. There are two basic routes by which the targeting ligands can be temporarily deactivated: (1) caged ligands, by using photocleavage groups to chemically cage the ligands; and (2) shielded ligands, by using molecular chains to physically shield the ligands [129]. Light irradiation can then activate the ligands by removing caging or shielding groups (Figure 11). Table 5 shows some examples of light-targeting through the activation of targeting ligands.

### 4.2. Light-Targeting through Particle Size Reduction

**Figure 12:**
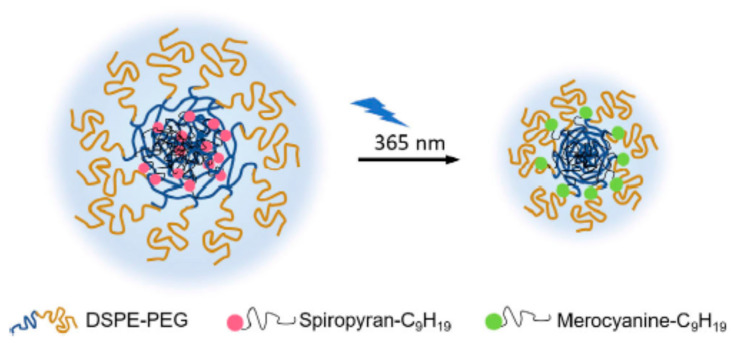
Light-targeting through a particle size reduction strategy. The neutral spiropyran changes to the zwitterionic merocyanine following UV irradiation, resulting in nanoparticle rearrangement and size reduction. Figure adapted with permission from [129].

Nanocarrier size can influence tumor accumulation and penetration capacity in the tumor microenvironment. In general, nanocarriers with an average size below 100 nm are preferred to achieve deep tissue penetration, site-specific release, and targeted drug delivery [136]. Light can be used to reduce the particle size of nanocarriers, thereby enhancing tissue penetration and increasing tumor-targeting efficiency. For instance, Tong et al. [137] designed light-responsive nanohybrids made of PEGylated lipid and alkyl chain-conjugated spiropyran that were capable of shrinking upon UV irradiation and thereby achieved deep tissue penetration. Upon UV irradiation at 365 nm, the hydrophobic spiroyran transformed from the neutral spiroyran to the zwitterionic merocyanine, resulting in the nanohybrid inner cores’ structural rearrangement (Figure 12). The particle size reduction promoted the release of the encapsulated drug at its target site.

Interestingly, light-targeting through particle size reduction can also be used for the effective targeting of intracellular organelles, e.g., Golgi vesicles, lysosomes, and nuclei [129]. However, in order to target the nucleus, the nanocarrier system should be able to penetrate the cell nucleus via nuclear pores, which have pore sizes of 9–40 nm. Since nanocarriers with average sizes of less than 10 nm are quickly removed from the blood stream by renal filtration [138], Qiu et al. [139] developed light-responsive gold nanoparticles containing doxorubicin as an anticancer drug and cell-type-specific internalizing aptamers to effectively target the cell nucleus. Upon NIR irradiation at 808 nm, the self-assembled structures of nanoparticles were disassembled due to the photothermal effect of NIR light, leading to the release of the drug from the nanocarrier system.

### 4.3. Light-Targeting through Blood Vessel Disruption

**Figure 13:**
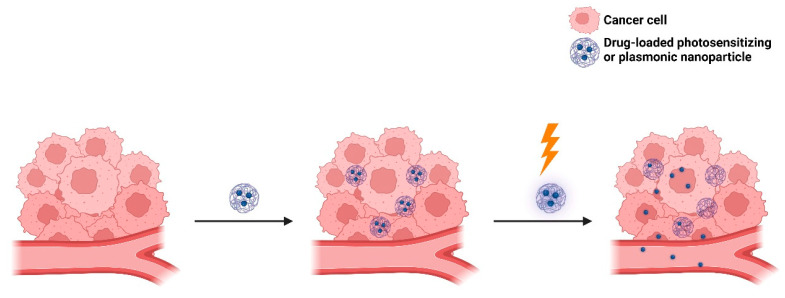
A schematic representation of the enhanced intra-tumoral accumulation of nanocarriers using PDT or PTT.

Light-targeting through blood vessel disruption improves EPR-mediated drug targeting to tumors [140]. This strategy comprises three main approaches: photodynamic therapy (PDT), photothermal therapy (PTT), and photoimmunotherapy (PIT). As mentioned earlier, PDT involves the use of dyes that are excited to a higher energy singlet state, from which they perform intersystem crossing to a triplet state that is suitable for energy transfer to molecular oxygen (^3^O_2_), forming singlet oxygen (^1^O_2_) or other reactive oxygen species (ROS) [141,142]. PDT can damage tumor endothelial cells, increase vascular permeability, and thereby improve the EPR effect and drug delivery to the tumor site [141,142]. In turn, PTT utilizes light to generate heat from plasmonic nanoparticles to kill the tumor cell (Figure 13). In the case of PIT, it utilizes antibody–photosensitizer conjugates that precisely bind to cells in the immediate perivascular space [143]. PIT can damage tumor cells through photosensitization, increase vascular permeability, and thereby improve drug delivery at the tumor site. For example, Sano et al. [144] developed panitumumab–photosensitizer conjugates. Upon IR irradiation at 690 nm, a high leakage rate of nanoconjugates (10–200 nm) into the A431 (human epidermoid carcinoma) cell line was observed clearly, indicating the potential of PIT to enhance nanodrug delivery in tumors.

## 5. Light-Responsive Liposomes for Drug Delivery

Light-responsive liposomes have been introduced as a smart nanocarrier for the spatiotemporal control of drug release. Hence, the triggering feature of light-responsive liposomes greatly enhanced the therapeutic efficacy and minimized the possible side effects of therapeutics [103,106]. Light-triggered drug release from liposomes can occur through two main approaches: (1) the photo-destabilization or disassembly of the liposomal structure, and (2) the light absorption of metallic nanoparticles. In the case of the photo-destabilization or disassembly of the liposomal structure, different photosensitizers can cause membrane destabilization and permeabilization. They can be strategically incorporated into lipid bilayers. The modification of phospholipids can occur at potential sites, e.g., the hydrophilic polar heads, glycerol backbone, and fatty acyl side chains [145,146]. The drug release can occur through one of the previously mentioned mechanisms of light-triggered drug release from liposomes (Figure 6). In the case of incorporating metallic nanoparticles into the liposomal structure, these nanoparticles can be localized within the lipid bilayers, on the surface of liposomes, aggregate in the core of liposomes, or be free in the aqueous or buffer compartment [147,148]. Upon irradiation of the liposomes, nanoparticles convert the absorbed photon energy to thermal energy, leading to the instability of the liposomal structure [147,148].

### 5.1. Formulation, Design, and Optimization

To design optimal liposomes for drug delivery, it is important to consider certain factors within the liposomal structure, such as size, lamellarity, surface charge, bilayer fluidity, and liposomal surface modification. To endow liposomes with photoactivation properties, additional photophysical properties should be considered, such as the type and concentration of photoswitch embedded in lipid bilayer membranes, the photosensitizer hydrophobicity and membrane localization (i.e., photosensitizer–membrane interactions), the spectral and photosensitizing properties of the photosensitizer used, the wavelength of the selected light source, and its penetration depth. Most of these parameters were discussed earlier in this review, while other parameters will be discussed below in detail.

#### 5.1.1. Liposomal Size

Liposome size is considered one of the most important parameters in the design of optimal liposomes, since liposome biodistribution, tumor accumulation, and clearance are primarily dependent on liposome size. Moreover, liposome size affects the immunogenicity and plasma half-life of the liposomal drug nanocarrier, thus affecting the drug circulation time and tumor targeting. In addition, the size of liposomes influences their endocytosis, thereby affecting their intracellular distribution and drug activity [149]. The excretion of liposomes is mainly through the liver and kidney, where kidney elimination is faster than liver elimination. Liposomes with small particle sizes (<5 nm) are mainly eliminated through the kidney, while liposomes with large particle sizes (up to 100 nm) are mainly eliminated through the liver due to the fact that larger particle sizes can be easily engulfed by macrophages [150,151]. In fact, liposomes with small particle sizes are not easily cleared by the phagocytes of mononuclear phagocyte systems (MPSs), but they can be rapidly eliminated by the kidney. Thus, liposomes with particle sizes between 5 and 100 nm have a longer circulation time [150,152]. Targeted liposomes with a small particle size and narrow size distribution leverage the advantages of the pathophysiological characteristics of the tumor microenvironment, including large fenestrations in the tumor vasculature (200–400 nm in size), elevated levels of inflammatory factors that retain tumor vasculature leakiness, and the lack of functional lymphatic drainage in the tumor cells. These factors together constitute the phenomenon of the EPR effect [22,23]. As the liposome size increases, the EPR effect is expected to decrease. Thus, to benefit from the EPR effect and to avoid clearance by the MPS, liposomes for targeted drug delivery are typically <200 nm [22,23]. On the other hand, liposomal size and lamellarity affect liposome distribution and circulation time. According to size and lamellarity, multilayered liposomes have more barriers to cross than single-layered liposomes, and therefore, semipermeable drugs may be retained for longer [153,154]. In addition, the adherence of serum proteins to liposomes may disturb lipid packaging so as to make unilamellar liposomes leak and lose their cargos, while in the case of multilayered liposomes, the protein adsorption will occur but only to the outer membrane, while the inner membrane will be shielded and thus they will retain their cargos longer [153,154]. Liposomal size and lamellarity also affect drug encapsulation and release. For instance, MLVs have a low aqueous volume for encapsulation; therefore, they are preferable for the encapsulation of bilayer-interacting hydrophobic drugs and less preferable for hydrophilic drugs. For hydrophilic drugs, LUVs are preferable owing to the large aqueous volume available for encapsulation, whereas SUVs have very low entrapment efficiency owing to their low encapsulation volume. Therefore, for hydrophilic drugs, encapsulation seems to be in the following order: MLV < SUV < LUV [154,155].

#### 5.1.2. Surface Charge

The surface charge of liposomes is primarily dependent on the head group of the liposomal phospholipid, and it can be either negative, neutral, or positive. Negatively charged phospholipids or anionic liposomes are easily recognized by macrophages, and they can enter cells via endocytosis at a higher rate than neutral phospholipids, resulting in a shorter circulation time. On the other hand, positively charged phospholipids or cationic liposomes are rapidly cleared from the blood circulation through complement activation or opsonization. Thus, anionic liposomes are preferred for preclinical and clinical studies and are common to most FDA- and EMA-approved liposomal formulations. In general, the zeta potential of liposomes (<−30 mV or >+30 mV) is considered physically stable due to electrostatic repulsive forces [156].

### 5.2. Light Source Selection

Various light sources have been developed for diagnostic and therapeutic applications. Among these light sources are lasers, light-emitting diodes (LEDs), and X-rays. Lasers have been widely used for the treatment of retinal diseases, such as diabetic retinopathy, retinal tears or detachment, and retinal vascular diseases, owing to their superior monochromatic performance and their ability to centralize into a coherent beam with extremely high energy density in a very short period of time, which can be a few microseconds or even milliseconds [157,158]. LEDs are another important light source that have been widely used in diagnostic and therapeutic medicine. Compared to lasers, LEDs offer several advantages and unique properties, such as the following: (1) the handling of LEDs is relatively easy and safe since it does not require a high voltage; and (2) they can be integrated into digital systems, facilitating complex light-setting programs such as varying spectral composition over the course of a phototherapy or during different treatment stages [159,160]. X-rays are another form of ionizing radiation that have been widely used in diagnostic and clinical applications, such as radiotherapy. The high energy of X-ray radiation leads to DNA damage either through ionization or through the generation of cytotoxic free radicals that result in apoptosis [161]. Recently, X-ray irradiation has been reported to trigger drug release from liposomes as well as in combinational treatments synergized with other therapies [162].

Light is electromagnetic (EM) radiation with a wide range of wavelengths. The EM radiation commonly used in photo-responsive liposomes is UV (200–400 nm), visible (400–700 nm), and NIR (700–1000 nm) light [64,67]. UV light is the most widely used radiation because it can provide sufficient energy to trigger most photochemical reactions (e.g., isomerization, cleavage crosslinking, or de-crosslinking reactions) [66,68]. Nevertheless, the use of UV light is accompanied by some serious disadvantages related to tissue penetration depth and phototoxicity. Therefore, the potential use of UV-light-responsive liposomes in clinical applications is very limited. On the other hand, visible light can generate higher energy; however, there are some disadvantages limiting its therapeutic uses, such as the limited tissue penetration depth of visible light and the ability of endogenous fluorophores, such as melanin and hemoglobin, to absorb visible light, which dramatically affects the tumor tissue penetration capability of visible light [64,67]. On the contrary, NIR light achieves deeper tissue penetration due to the minimal attenuation and refraction by endogenous chromophores and biomolecules [66,67,68]. Although NIR light possesses the advantages of deeper tissue penetration and less damage to normal cells, only a few compounds are able to respond to NIR light directly, owing to the low energy of NIR light, which is insufficient to trigger photochemical reactions [66,67,68]. To solve this problem, nanocarriers that can convert incident NIR light to UV light have been developed. The resulting UV light can allow photochemical reactions, resulting in effective drug release. The conversions of lower energy NIR photons to higher energy UV photons encompass the processes of TPA [119,120,121] and UCNPs [123,124,125]. To conclude, the most suitable light wavelength for in vivo approaches is between 650 and 900 nm, with tissue penetration depth at the millimeter scale, referred to as the “first NIR window” or “optical window”.

#### 5.2.1. Light Penetration Depth

The selection of the proper wavelength of the light source determines the tissue penetration depth. Therefore, it is important to select a suitable light source with an optimal wavelength to induce tumor-specific photoactivation. In general, penetration depth increases along with wavelengths, which can range from a few hundred microns of UV lights to >5 mm of NIR lights [66,67,163]. UV lights are preferred for the treatment of early-stage (superficial) cutaneous cancers, while NIR lights are preferred for late-stage (deep) cutaneous cancers [66,67,164].

#### 5.2.2. Photodamage

The excessive use of UV light causes photolesions that distort the DNA double helix structure and thus lead to DNA damage, which eventually results in cancer [165]. In turn, the long exposure to visible light induces cell receptor and retinal photodamage [166]. On the other hand, NIR light possesses the advantages of deeper penetration depth in tissues, as well as no or less DNA damage and genotoxicity [167]. Therefore, most of the current research on light-responsive liposomes is focused on using NIR light.

## 6. Dual-Targeting Stimuli-Triggered Liposomes

In the past few decades, researchers have attempted to make the liposomal nanocarriers more tumor-specific and effective by combining two or more stimuli in a single drug-loaded vehicle, leading to the development of dual-targeted liposomes. Dual-targeted liposomes have several advantages over conventional liposomes, such as targeting two or more receptors, a better tumor cellular internalization, releasing the encapsulated drugs with higher efficiency and accuracy, and avoiding normal tissue toxicity [56,168]. By combining two different stimuli in one liposomal formulation, site-specific and multistage targeting can be precisely achieved.

### 6.1. Light/pH Dual-Responsive Liposomes

As a selected example of combining light with pH stimuli, Kong et al. [169] developed a biodegradable multifunctional nanoplatform of photothermal-responsive calcium carbonate particles coated with pH-responsive acetalated dextran and phospholipid, abbreviated AuNR@CaCO3@POPC-AcDX, as a novel nanoplatform for the incorporation of both hydrophilic and hydrophobic molecularly targeted therapeutics with high EE% and LC%. The AuNR@CaCO3@POPC-AcDX hybrid nanoplatform was effective in the growth inhibition of cancer cells with specific molecular targeting and overcame multidrug resistance and possible adverse drug reactions. The photothermal effect promoted therapeutic ultrafast release and speedy cancer cell death. Another interesting selected example is Chen et al. [170], who designed a pH-sensitive charge-conversional and NIR-responsive bubble-generating liposomal complex named bubble-generating thermosensitive liposomes (BTSL) made of cypate, doxorubicin, and poly(methacryloyl sulfadimethoxine) for synergetic thermo-chemotherapy for tumors. The cationic liposomes containing cypate, doxorubicin, and NH_4_HCO_3_ were first shielded by pH-sensitive poly(methacryloyl sulfadimethoxine) through electrostatic interaction at physiological pH 7.4. Then, at pH 6.5 (reflecting the tumor microenvironment), PSD was de-shielded; as a result, the liposomal formulation displayed a pH-sensitive charge reversal capability. The doxorubicin was released from PSD/DOX/Cypate-BTSL through NIR irradiation. After NIR irradiation, the hyperthermia induced by cypate was capable of producing CO_2_ bubbles owing to the decomposition of NH_4_HCO_3_, resulting in a robust drug release. In 4T1 breast cancer cells, PSD/DOX/Cypate-BTSL improved cellular uptake and cytotoxicity.

### 6.2. Light/Temperature Dual-Responsive Liposomes

As a selected example of combining light with temperature, You et al. [171] developed a novel liposomal formulation containing cisplatin, indocyanine green (ICG), and CJM126 mixed with a cholesterol derivative (CJM-Chol) for the purpose of synergistic chemo-photothermal therapy. Liposomes were prepared using the thin-film hydration method. The prepared liposomes showed a uniform diameter of 103.8 nm and a good polydispersity of 0.195. Irradiation with NIR induced photothermal conversion, which triggered rapid drug release from liposomes. Outstandingly, the light-induced heat-initiated drug release at a temperature > 42 °C accelerated the drug release and made it more controllable. Moreover, the prepared liposomes showed a significantly excellent inhibitory effect (3.05% cell viability in 24 h) on MDA-MB-231 breast cancer cells when irradiated with NIR light as compared with free cisplatin (28.41%) or treatment without NIR (11.24%), which was significantly superior to chemotherapy or photothermal therapy alone. Another interesting selected example is Luo et al. [172], who prepared gold nanoshell-coated oleanolic acid liposomes (GNOLs) mediated by chitosan. The GNOLs were spherical in shape with a uniform diameter size of 72.03 nm and a zeta potential of 20.7 mV, which made them more likely to be accumulated at the tumor site. The GNOLs exhibited a slow release of oleanolic acid at pH 7.4 and a robust release at pH 5.5, which was favorable for tumor-triggered drug release. Under NIR irradiation, hyperthermia was produced by activated gold nanoshells, which triggered drug release from the liposomes by modulating the gel-to-liquid crystalline phase transition of the liposomes. On account of the photothermal effect of gold nanoshells and the thermal sensitivity of the lipid bilayers of liposomes, the lipid coat was destabilized after NIR irradiation, and a robust drug release was achieved. Because of the pH-responsive nature of the cationic polymer chitosan, the encapsulated drug was able to identify drug targets easily and achieve intracellular tumor site-specific drug release. The novel gold nanoshell-coated oleanolic acid liposomes mediating tumor therapy represented a potentially important advancement in chemo-photothermal therapy.

## 7. Challenges in Light-Triggered Drug Release from Liposomes

Despite all the advantages of light-responsive liposomes, there are still challenges associated with UV and NIR light that limit their use and application. For instance, the use of UV light is often associated with a high risk of tissue damage that is not limited to tumor tissues but also affects the surrounding normal tissues, and thereby may eventually lead to therapeutic failure and incomplete tumor eradication [64,65,66,67,68]. Though NIR is more preferable for drug delivery and release, the low-energy wavelength light of NIR may not be sufficient to induce photochemical effects [64,65,66,67,68]. Other emerging challenges in light-based therapy include increasing the tissue penetration depth of incident light, increasing the tumor selectivity of the used photosensitizer, improving the efficacy and efficiency of photo-responsiveness, and optimizing the switch-on and switch-off transitions [173].

On the other hand, although remarkable progress has been made during the past few decades in the design and development of nanocarrier systems, there are still some challenges in their clinical research. For example, the size of most nanocarriers ranges from 10 to 100 nm, owing to some technical limitations related to their preparation methods [174,175]. It is worth mentioning that nanocarriers with sizes ≥ 200 nm are primarily accumulated in extracellular spaces, while nanocarriers with sizes ≤ 10 nm can easily be filtered out [174,175]. The major challenges in the clinical research of nanocarriers lie in finding the right target for disease diagnosis, the proper drug for disease treatment, and the most suitable targeting strategy for site-specific drug delivery [174,175]. In the matter of light-responsive liposomes, the methodological complexity of light-responsive liposomes hinders their industrial scale-up; thus, new industry-oriented methods are necessary for the synthesis and application of smart-generation light-responsive liposomes. Moreover, the lack of clinical data related to the safety and efficacy of light-responsive liposomes in vivo greatly limits their widespread therapeutic use; therefore, more clinical trial data are necessary to further advance the clinical significance of light-responsive liposomes.

## 8. Emerging Trends and Future Prospects

The clinical applications of light-responsive liposomes are limited by light penetration depth; however, new advancements in light technology, such as fiber optic endoscopy (FOE), have enabled the temporary placement of optics to target deep tissues. Moreover, novel strategies in photo-responsiveness, such as the development of photocleavable groups that are capable of either being activated by long-wavelength lights or by efficient up-conversion systems, or that contain photo-protecting groups with red-shifted absorption, have led to improved tissue penetration depth [176,177,178]. With regard to photosensitizers, the modification of the photosensitizer core by substitutes led to wavelength-shifting to the blue-green region [179]. Moreover, high-efficiency triplet-triplet annihilation (TTA) up-conversion systems were fabricated to tolerate large stock shifts [180]. For example, Huang et al. [180] designed a TTA up-conversion system of long-wavelength light from the far-red (600–670 nm) to the deep-blue (410–500 nm) region for the efficient activation of photo-responsive molecules. On the other hand, the combination of light with other triggers was able to increase the triggering precision [181]. For instance, Lin et al. [182] developed a light-activated hypoxia-responsive drug delivery system in which the encapsulated drug was bonded to 7-aminocoumarin through a photocleavage bond. Additionally, nitroimidazole, an electron acceptor, was coupled to 7-aminocoumarin in order to prevent bond breakage upon irritation, owing to photo-induced electron transfer (PET) phenomena. The nitro-to-amino hypoxia-specific reduction converted nitroimidazole to aminoimidazole, leading to the loss of the PET effect. Once at the tumor site and under hypoxic conditions, the drug was released from the coumarin conjugate following light irradiation. This design increased tumor-targeting selectivity and reduced the toxicity to healthy tissues surrounding the tumor.

## 9. Conclusions

Tuning the release and activity of drug nanocarriers via the application of light represents an innovative technology and approach in the field of drug delivery since it allows for optimum spacing, precise bonding timing, and accurate positioning between drugs and their receptors, and therefore delineates the optimal configuration of the nanocarrier system with enhanced physicochemical and photophysical characteristics. Photo-responsive nanocarriers have recently received increasing attention as smart drug nanocarrier systems, aiming to deliver drugs to target specific tumor sites with high spatial and temporal control over drug release. These systems mainly utilize nonionizing radiation and are primarily composed of biodegradable polymers. The physicochemical approaches that endow nanocarriers with photoresponsivity are categorized into three different classes: (1) photochemically triggered, where the absorbed light energy is enough to simply break up covalent bonds or through a photochemical reaction; (2) photoisomerization, where the excess energy induces structural changes; and (3) photothermal, where the absorbed photon energy is dissipated via vibrational motion. Liposomes are popular nanocarriers used in encapsulating both hydrophilic and lipophilic drugs for targeted delivery. Liposomes are considered one of the healthiest, safest, and most effective nanocarriers developed so far. They are composed of naturally occurring substances that can be easily metabolized inside the human body, so they can be regarded as biodegradable, biocompatible, safe, and non-toxic drug vehicles [183]. Recent advances in liposomal drug delivery comprise (1) long-circulating (sterically stabilized); (2) remote loading of drugs into liposomes by pH and ion (ammonium or acetate) gradients; and (3) lipoplexes by the interaction of anionic nucleic acids or proteins with the surface of cationic liposomes [184,185]. These advances encompass the improvement of drug loading capacity and encapsulation efficiency, as well as the enhancement of drug pharmacokinetics, biodistribution, therapeutic efficacy, and the reduction of systemic side effects and toxicity. Liposomes are capable of targeted, specific modification, and stimuli-responsiveness, which make them more effective in cancer treatment. Their flexibility for surface modification by the addition of targeting moieties make liposomes more promising candidates. Targeting ligand surface-modified liposomes are often combined with different stimuli for better localized delivery and chemotherapeutic release with minimal systemic exposure and reduced toxicity. The progress from single-function to multifunctional-responsive liposomes has demonstrated huge therapeutic potential for targeted cancer therapy. The development of multifunctional liposomes with light-responsive properties sheds light on highly efficient combined cancer therapy.

## Figures and Tables

**Table 1:** Lipids used for the preparation of liposomes (data extracted from the Sigma-Aldrich Aldrich (Burlington, MA, USA) and Avanti Polar Lipids Alabaster, AL, USA) databases).

Lipid Name and CAS No.	Synonym	Molecular Formula	Chemical Structure
**Neutral**			
Cholesterol (CAS No.: 57-88-5)	---	C_27_H_46_O	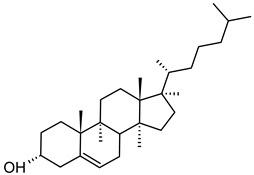
**Anionic**			
1,2-Dimyristoyl-*sn*-glycero-3-phosphoglycerol, sodium salt (CAS No.: 200880-40-6)	DMPG-Na	C_34_H_66_NaO_10_PNa	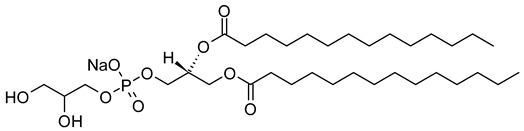
1,2-Dipalmitoyl-*sn*-glycero-3-phosphoglycerol, sodium salt (CAS No.: 200880-41-7)	DPPG-Na	C_38_H_74_NaO_10_PNa	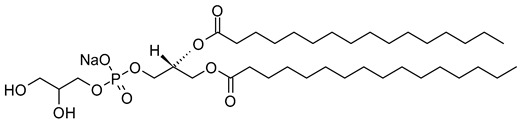
1,2-Distearoyl-*sn*-glycero-3-phosphatidylglycerol, sodium salt (CAS No.: 200880-42-8)	DSPG-Na	C_42_H_82_NaO_10_PNa	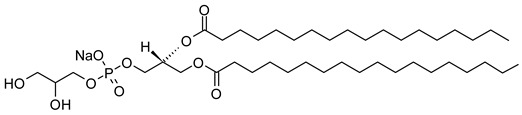
N-(Methoxypolyethylene glycol 5000 carbamoyl)-1,2-dipalmitoyl-*sn*-glycero-3-phosphatidylethanolamine, monosodium salt (CAS No.: 205494-72-0)	MPEG-5000-DPPE-Na	(C_2_H_4_O)*_n_*C_39_H_76_NO_10_PNa	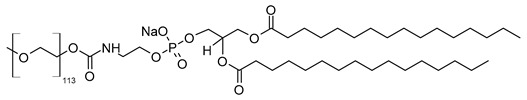
N-(Methoxypolyethylene glycol 2000 carbamoyl)-1,2-dipalmitoyl-*sn*-glycero-3-phosphatidylethanolamine, monosodium salt (CAS No.: 384835-61-4)	MPEG-2000-DPPE-Na	(C_2_H_4_O)*_n_*C_39_H_76_NO_10_PNa	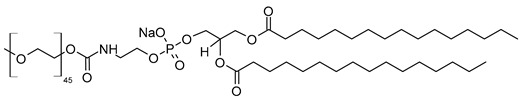
**Cationic**			
1,2-dioleoyl-3-trimethylanmmonium-propane (chloride salt) (CAS No.: 132172-61-3)	DOTAP	C_42_H_80_NO_4_Cl	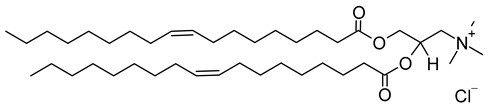
1,2-di-O-octadecenyl-3-trimethylammonium propane (chloride salt) (CAS No.: 104872-42-6)	DOTMA	C_42_H_84_ClNO_2_	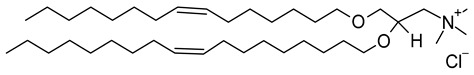
**Zwitterion**			
1,2-dimyristoyl-sn-glycero-3-phosphocholine (CAS No.: 18194-24-6)	DMPC	C_36_H_72_NO_8_P	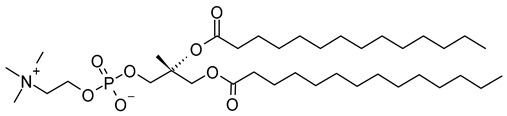
1,2-Dipalmitoyl-*sn*-glycero-3-phosphocholine (CAS No.: 63-89-8)	DPPC	C_40_H_80_NO_8_P	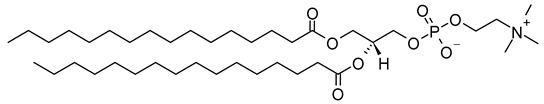
1,2-Distearoyl-*sn*-glycero-3-phosphoethanolamine (CAS No.: 1069-79-0)	DSPE	C_41_H_82_NO_8_P	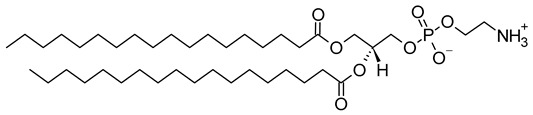
L-α-phosphatidylcholine, hydrogenated (soy) (CAS No.: 97281-48-6)	HSPC	C_42_H_84_NO_8_P	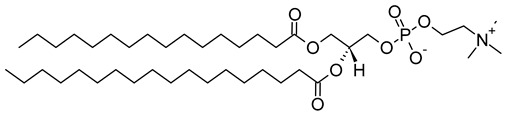
1-Palmitoyl-2-oleoyl-*sn*-glycero-3-phosphocholine (CAS No.: 26853-31-6)	POPC	C_42_H_82_NO_8_P	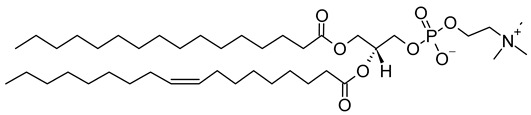
1,2-Dioleoyl-*sn*-Glycero-3-phosphocholine (CAS No.: 4235-95-4)	DOPC	C_44_H_84_NO_8_P	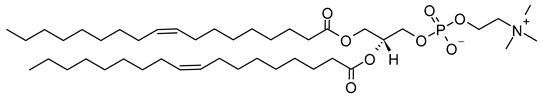
1,2-Distearoyl-*sn*-glycero-3-phosphocholine (CAS No.: 816-94-4)	DSPC	C_44_H_88_NO_8_P	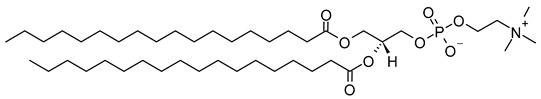
**Photoswitchable Lipids**			
1-stearoyl-2-[(E)-4-(4-((4-butylphenyl)diazenyl)phenyl)butanoyl]-*sn*-glycerol (CAS No.: 1985595-31-0)	18:0-PhoDAG	C_41_H_64_N_2_O_5_	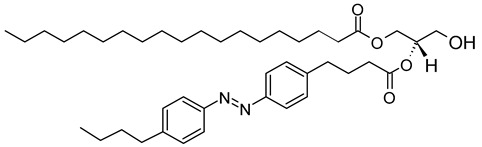
N-[(E)-4-(4-((4-butylphenyl)diazenyl)phenyl)butanoyl]-D-erythro-sphingosylphosphorylcholine (CAS No.: 2260670-56-0)	Azo SM	C_43_H_71_N_4_O_6_P	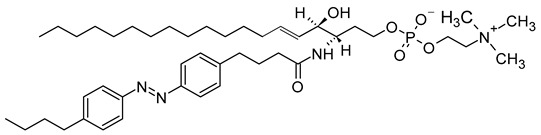
1-stearoyl-2-[(E)-4-(4-((4-butylphenyl)diazenyl)phenyl)butanoyl]-*sn*-glycero-3-phosphocholine (CAS No.: 2098674-45-2)	18:0-azo PC	C_46_H_76_N_3_O_8_P	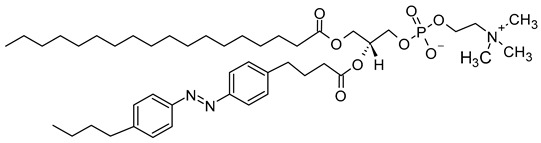

**Table 2:** FDA- and EMA-approved liposomal drug formulations [20,21].

Product Name	Approval Date	Product Description	Liposome Composition	Indication and Usage	Manufacturer
Doxil^®^	FDA: 1995 EMA: 1996	Doxorubicin encapsulated in stealth liposomes.	MPEG-DSPE, HSPC, cholesterol.	Ovarian cancer, AIDS-related Kaposi’s sarcoma.	Janssen Pharmaceuticals (Beerse, Belgium)
Abelcet^®^	FDA: 2005	Amphotericin B lipid complex injection.	DMPC, DMPG.	Invasive fungal infections.	Leadiant Biosciences, Inc. (Gaithersburg, MD, USA)
DaunoXome^®^	FDA: 1996 EMA: 2004	Daunorubicin encapsulated in liposomes.	DSPC, cholesterol.	Advanced HIV-associated Kaposi’s sarcoma.	Galen Ltd. (Craigavon, UK)
AmBisome^®^	FDA: 1997 EMA: 2006	Amphotericin B liposome for injection.	HSPC, cholesterol, DSPG, alpha tocopherol.	Cryptococcal meningitis in HIV-infected patients.	Gilead Sciences, Inc. (Foster City, CA, USA)
DepoCyt^®^	FDA: 1999 EMA: 2001	Cytarabine liposome injection.	Cholesterol, triolein, DOPC, DPPG.	Lymphomatous meningitis.	Pacira Pharmaceuticals, Inc. (Parsippany, NJ, USA)
Myocet^®^	FDA: 2000 EMA: 2000	Non-PEGylated liposomal doxorubicin.	Phosphatidylcholine, cholesterol.	Metastatic breast cancer in adult women.	Teva Pharmaceuticals (Tel Aviv, Israel)
Mepact^®^	FDA: 2001 EMA: 2009	A liposomal suspension of mifamurtide.	POPC, OOPS.	High-grade resectable non-metastatic osteosarcoma.	Takeda Pharmaceutical Company (Tokyo, Japan)
Exparel^®^	FDA: 2011 EMA: 2021	Bupivacaine liposome injectable suspension.	Cholesterol, DPPG, DEPC.	Postsurgical regional analgesia.	Pacira Pharmaceuticals, Inc. (Parsippany, NJ, USA)
Onivyde^®^	EMA: 2016	Irinotecan sucrosofate in PEGylated liposomes.	DSPC, cholesterol, MPEG-2000-DSPE.	Metastatic adenocarcinoma of the pancreas.	Laboratoires Servier (Servier) (Suresnes, France)
Vyxeos^®^	FDA: 2017	Cytarabine and daunorubicin liposome injection.	DSPC, DSPG, cholesterol.	Acute myeloid leukemia.	Jazz Pharmaceuticals plc (Dublin, Ireland)
Arikayce^®^	FDA: 2018 EMA: 2020	Amikacin liposome inhalation suspension.	Cholesterol, DPPC.	Non-tuberculous mycobacterial (NTM) lung infections.	Almac Pharma Services Ltd. (Athlone, Ireland)
Zolsketil^®^	EMA: 2022	Doxorubicin in PEGylated liposomes.	MPEG 2000-DSPE, HSPC, cholesterol.	Ovarian neoplasms, sarcoma, Kaposi, multiple myeloma.	Accord Healthcare S.L.U. (Barcelona, Spain)

**Table 3:** Examples of liposomes and their ligands used for active targeting.

Active Targeting Ligand	Encapsulated Drug	Preparation Method	Reference
**PEGylated Liposomes**			
mAbs (MM-302)	Doxorubicin	Thin-film hydration	[26]
mAbs (Sortagged anti-EGFR)	Doxorubicin	Ethanol injection	[27]
Folate	Oleuropein	Thin-film hydration	[28]
Folate	Rapamycin	Thin-film hydration	[29]
Folate	Arsenic trioxide	Thin-film hydration	[30]
Transferrin	Plumbagin	Thin-film hydration	[31]
Transferrin	Resveratrol	Thin-film hydration	[32]
Mannose	Chlorogenic acid	Thin-film hydration	[33]
cRGD	microRNA	Thin-film hydration	[34]
**Cationic Liposomes**			
Transferrin	Doxorubicin	Ethanol injection	[35]
mAbs (Herceptin)	Curcumin	Thin-film hydration	[36]
Aptamer (AS1411)	Paclitaxel and siRNA	Thin-film hydration	[37]

**mAbs**: Monoclonal antibodies; **MM-302**: An anti-HER2 mAb; **Sortagged anti-EGFR**: Sortase-A mediated CH–LPETG–mAb; **cRGD**: Cyclic arginine-glycine-aspartic acid peptide; **RNA**: Ribonucleic acid; **Herceptin**: An anti-HER2 mAb; **Aptamer (AS1411)**: A guanosine-rich oligonucleotide aptamer; **siRNA**: Small interfering RNA.

**Table 4:** Comparison between different types of stimuli.

Stimuli	Advantages	Limitations	References
Light	- Sequentially triggers multiple payloads. - High degree of spatiotemporal precision. - Operates over a broad spectrum of wavelengths.	- Low penetration for UV- and visible light. - Overexposure to UV/visible irradiation can cause serious health problems. - NIR penetrates tissues more deeply but with lower energy.	[64,65,66,67,68]
Heat	- Suitable for cancer cells, which are highly sensitive to hyperthermia. - Enhanced tumor vascular permeability. - Reduced hypoxic conditions.	- Risk of superficial tissue damage. - Difficult to spatially control hyperthermia at the tumor site.	[69,70]
pH	- Intrinsically safe and effective. - Highly sensitive and specific. - pH-sensitive strategies allow site-specific drug delivery.	- Slow kinetics of drug release. - A small change in the pH can cause the instability of the nanocarrier system.	[71,72]
Electrical fields	- Iontophoresis devices generate safe levels of electrical fields with different strengths. - Readily accessible in the clinic.	- Risk of healthy tissue damage. - Electro-responsiveness is greatly affected by several environmental factors, such as the composition of aqueous medium, and the types and concentrations of electrolytes.	[73,74]
Magnetic fields	- Magnetic-controlled drug release with high precision. - Iron oxide materials are commonly used in magnetic-triggered drug delivery due to their biocompatibility.	- Complex installation and operating system. - Potential toxicity from metals. - Difficult to focus alternating magnetic field.	[75,76]
Ultrasound waves	- Non-ionizing safe radiation with high penetration. - Minimal safety risks with low intensity, short exposure, and a high degree of spatiotemporal precision.	- Ultrasound-responsive medium (gas/PFC) is required. - Risk of healthy tissue damage. - Drug-carrier instability issues.	[77,78]

**Table 5:** Examples of light-targeting though the activation of targeting ligands of liposomes.

Encapsulating Drug	Ligand Type	Caging/Shielding Group	Irradiation Source	Reference
siRNA	CPP/PCP	PEG	NIR	[130]
Vinorelbine bitartrate	PSP/NGR	PEG	NIR	[131]
-----	TAT	PEG	UV	[132]
siRNA	pcCPP/NGR	PEG	NIR	[133]
5(6)-carboxyfluorescein	AMP (BTL)	ε-amino group of the Lys in TL	UV	[134]
Paclitaxel	Folate	o-nitrobenzylamine	UV	[135]

**siRNA**: Small interfering RNA; **CPP**: Cell-penetrating peptide (CGRRMKWKK); **PCP**: Photolabile-caged peptide (CGRRMKPGWKPGKPG); **PSP**: Photosensitive peptide (CGRRMKPGWKPGKPG); **NGR**: Asparagine–glycine–arginine (CYGGRGNG); **TAT**: Transactivating transcriptional activator (YGRKKRRQRRRG); **PEG**: Polyethylene glycol; **pcCPP**: Photolabile-caged cell-penetrating peptide; **AMP**: Antimicrobial peptide, **BTL**: Bhcmoc-temporin L.

## Data Availability

Not applicable.

## Abbreviations

**Abbreviation**	**Definition**
ABC	Accelerated blood clearance
ALA	5-aminolevulinic acid
Azo SM	N-[(E)-4-(4-((4-butylphenyl)diazenyl)phenyl)butanoyl]-D-erythro-sphingosylphosphorylcholine
BHA	4-butylazobenzene-4-hexyloxy-trimethyl-ammoniumtrifluoro-acetate
BHA-cur-lipo	BHA-curcumin-liposomes
Bhc	6-bromo-7-hydroxy-4-hydroxycoumarin
BTSL	Bubble-generating thermosensitive liposomes
CHEMS	Cholesteryl hemisuccinate
CJM-Chol	CJM126 mixed with cholesterol derivative
CMA	7-(2-methacryloyloxyethoxy)-4-methylcoumarin
CMC value	Critical micelle concertation value
P(MEOMA-co-CMA)	Coumarin-containing poly(2-(2-methoxyethoxy)ethyl methacrylate)
CPPs	Cell-penetrating peptides
CPT	Chemophototherapy
CTX	Cabazitaxel
CTX-PoP-Lip	Cabazitaxel-loaded porphyrin-phospholipid liposomes
CuAAC	Copper-catalyzed azide alkyne cyclo-addition
DNQ	2-diazo-1,2-naphthoquinone
DMPC	1,2-dimyristoyl-*sn*-glycero-3-phosphocholine
DMPG-Na	1,2-Dimyristoyl-*sn*-glycero-3-phosphoglycerol, sodium salt
DNA	Deoxyribonucleic acid
DOPC	1,2-Dioleoyl-*sn*-glycero-3-phosphocholine
DOPE	Dioleoyl-phosphatidylethanolamine
DOTMA	1,2-di-O-octadecenyl-3-trimethylammonium propane (chloride salt)
DOTAP	1,2-dioleoyl-3-trimethylanmmonium-propane (chloride salt)
DPPC	1,2-dipalmitoyl-*sn*-glycero-3-phosphocholine
DPPG-Na	1,2-Dipalmitoyl-*sn*-glycero-3-phosphoglycerol, sodium salt
DSPC	1,2-distearoyl-*sn*-glycero-3-phosphocholine
DSPE	1,2-Distearoyl-*sn*-glycero-3-phosphoethanolamine
DSPG-Na	1,2-Distearoyl-*sn*-glycero-3-phosphatidylglycerol, sodium salt
EE%	Percent encapsulation efficiency
EGFR	Epidermal growth factor receptor
EM	Electromagnetic
EMA	European Medicines Agency
EMC	Electronic Medicines Compendium
EPR	Enhanced permeability and retention
Fab	Antigen-binding fragment
FDA	U.S. Food and Drug Administration
FOE	Fiber optic endoscopy
FR	Folate receptor
GNOLs	Gold nanoshell-coated oleanolic acid liposomes
HLB	Hydrophilic-lipophilic balance
HSPC	L-α-phosphatidylcholine, hydrogenated (soy)
ICG	Indocyanine green
IgM	Immunoglobulin M
IR	Infrared
LC%	Percentage of loading capacity
LEDs	Light-emitting diodes
LUVs	Large unilamellar vesicles
MAA	Methacrylic acid
mAbs	Monoclonal antibodies
MAL	Methyl aminolevulinate
MC	Merocyanine
MEO_2_MA	2-(2-methoxyethoxy)ethyl methacrylate
MLVs	Multilamellar large vesicles
MPEG-2000-DPPE-Na	*n*-(methoxypolyethylene glycol 2000 carbamoyl)-1,2-dipalmitoyl-*sn*-glycero-3-phosphatidylethanolamine, monosodium salt
MPEG-5000-DPPE-Na	*n*-(methoxypolyethylene glycol 5000 carbamoyl)-1,2-dipalmitoyl-*sn*-glycero-3-phosphatidylethanolamine, monosodium salt
MPS	Mononuclear phagocyte systems
m-THPC	Meta-tetrahydroxy phenyl cholorin
NIR	Near-infrared
ONB	o-nitrobenzyl
PC	Phosphatidylcholine
PDT	Photodynamic therapy
PEG	Polyethylene glycol
PEG-PLEA	Poly(ethylene glycol)-poly(lactic acid-ethanolic acid)
PEG-PMI-DTE	PEGylated perylenemonoimide-dithienylethene
PEO	Polyethylene oxide
PET	Photo-induced electron transfer
PIT	Photoimmunotherapy
Pheo-a	Pheophorbide-a
PNB	Poly (4,5-dimethoxy-2-nitrobenzyl methacrylate
POPC	1-Palmitoyl-2-oleoyl-*sn*-glycero-3-phosphocholine
PTT	Photothermal therapy
Pyro-a	Pyropheophorbide-a
RES	Reticuloendothelial system
RNA	Ribonucleic acid
ROS	Reactive oxygen species
SC-CO_2_	Supercritical carbon dioxide
scFv	Single-chain variable fragment
SP	Spiropyran
SPR	Surface plasmon resonance
SP-to-MC	Spiropyran-to-merocyanine
SPTPC	Spiropyran-containing triazole-phosphatidylcholine
SUVs	Small unilamellar vesicles
TFR	Transferrin receptor
TPA	Two-photon absorption
TPC	Triazole-phosphatidylcholine
TTA	Triplet–triplet annihilation
UCNPs	Up-Converting nanoparticles
uPA	Urokinase-plasminogen activator
UV	Ultraviolet
VPTT	Volume-phase transition temperature
^1^O_2_	Singlet oxygen
^3^O_2_	Molecular oxygen
18:0-Azo PC	1-stearoyl-2-[(E)-4-(4-((4-butylphenyl)diazenyl)phenyl)butanoyl]-*sn*-glycero-3-phosphocholine (CAS No.: 2098674-45-2)
18:0-PhoDAG	1-stearoyl-2-[(E)-4-(4-((4-butylphenyl)diazenyl)phenyl)butanoyl]-*sn*-glycerol

## References

[1] Bangham A.D., Standish M.M., Watkins J.C. (1965). Diffusion of univalent ions across the lamellae of swollen phospholipids. J. Mol. Biol..

[2] Nsairat H., Khater D., Sayed U., Odeh F., Al Bawab A., Alshaer W. (2022). Liposomes: Structure, composition, types, and clinical applications. Heliyon.

[3] Chiang C.L., Cheng M.H., Lin C.H. (2021). From nanoparticles to cancer nanomedicine: Old problems with new solutions. Nanomater.

[4] Langer R. (1976). Polymers for the sustained release of proteins and other molecules. Nature.

[5] Le Saux S., Aubert-Pouëssel A., Ouchait L., Mohamed K.E., Martineau P., Guglielmi L., Devoisselle J.M., Legrand P., Chopineau J., Morille M. (2021). Nanotechnologies for intracellular protein delivery: Recent progress in inorganic and organic nanocarriers. Adv. Ther..

[6] Saleh T.A. (2020). Nanomaterials: Classification, properties, and environmental toxicities. Environ. Technol. Innov..

[7] Bangham A.D., Horne R.W. (1964). Negative staining of phospholipids and their structural modification by surface-active agents as observed in the electron microscope. JMB.

[8] Bangham A.D. (1972). Lipid bilayers and biomembranes. Annu. Rev. Biochem..

[9] Laouini A., Jaafar-Maalej C., Limayem-Blouza I., Sfar S., Charcosset C., Fessi H. (2012). Preparation, characterization and applications of liposomes: State of the art. J. Colloid Sci. Biotechnol..

[10] Agiba A.M., Nasr M., Abdel-Hamid S., Eldin A.B., Geneidi A.S. (2018). Enhancing the intestinal permeation of the chondroprotective nutraceuticals glucosamine sulphate and chondroitin sulphate using conventional and modified liposomes. Curr. Drug Deliv..

[11] Li J., Wang X., Zhang T., Wang C., Huang Z., Luo X., Deng Y. (2015). A review on phospholipids and their main applications in drug delivery systems. Asian J. Pharm. Sci..

[12] Alinaghi A., Rouini M.R., Daha F.J., Moghimi H.R. (2014). The influence of lipid composition and surface charge on biodistribution of intact liposomes releasing from hydrogel-embedded vesicles. Int. J. Pharm..

[13] Moghimi S.M., Szebeni J. (2003). Stealth liposomes and long circulating nanoparticles: Critical issues in pharmacokinetics, opsonization and protein-binding properties. Prog. Lipid Res..

[14] Marzban E., Alavizadeh S.H., Ghiadi M., Khoshangosht M., Khashayarmanesh Z., Abbasi A., Jaafari M.R. (2015). Optimizing the therapeutic efficacy of cisplatin PEGylated liposomes via incorporation of different DPPG ratios: In vitro and in vivo studies. Colloids Surf. B.

[15] Nicolosi D., Cupri S., Genovese C., Tempera G., Mattina R., Pignatello R. (2015). Nanotechnology approaches for antibacterial drug delivery: Preparation and microbiological evaluation of fusogenic liposomes carrying fusidic acid. Int. J. Antimicrob. Agents.

[16] Nakhaei P., Margiana R., Bokov D.O., Abdelbasset W.K., Jadidi Kouhbanani M.A., Varma R.S., Marofi F., Jarahian M., Beheshtkhoo N. (2021). Liposomes: Structure, biomedical applications, and stability parameters with emphasis on cholesterol. Front. Bioeng. Biotechnol..

[17] Chaves M.A., Baldino L., Pinho S.C., Reverchon E. (2022). Supercritical CO_2_ assisted process for the production of mixed phospholipid nanoliposomes: Unloaded and vitamin D_3_-loaded vesicles. J. Food Eng..

[18] Campardelli R., Baldino L., Reverchon E. (2015). Supercritical fluids applications in nanomedicine. J. Supercrit. Fluids.

[19] Trucillo P., Campardelli R., Reverchon E. (2017). Supercritical CO_2_ assisted liposomes formation: Optimization of the lipidic layer for an efficient hydrophilic drug loading. J. CO2 Util..

[20] US FDA Orange Book: Approved Drug Products with Therapeutic Equivalence Evaluations. https://www.fda.gov/drugs/drug-approvals-and-databases/approved-drug-products-therapeutic-equivalence-evaluations-orange-book.

[21] EMC Electronic Medicines Compendium: Approved and Regulated Prescribing and Patient Information for Licensed Medicines. https://www.medicines.org.uk/emc#gref.

[22] Danhier F., Feron O., Préat V. (2010). To exploit the tumor microenvironment: Passive and active tumor targeting of nanocarriers for anti-cancer drug delivery. J. Control. Release.

[23] Iyer A.K., Khaled G., Fang J., Maeda H. (2006). Exploiting the enhanced permeability and retention effect for tumor targeting. Drug Discov. Today.

[24] Alavi M., Hamidi M. (2019). Passive and active targeting in cancer therapy by liposomes and lipid nanoparticles. Drug Metab. Pers. Ther..

[25] Bae Y.H., Park K. (2011). Targeted drug delivery to tumors: Myths, reality and possibility. J. Control. Release.

[26] Dumont N., Merrigan S., Turpin J., Lavoie C., Papavasiliou V., Geretti E., Espelin C.W., Luus L., Kamoun W.S., Ghasemi O. (2019). Nanoliposome targeting in breast cancer is influenced by the tumor microenvironment. Nanomed. Nanotech. Biol. Med..

[27] Wöll S., Dickgiesser S., Rasche N., Schiller S., Scherließ R. (2019). Sortagged anti-EGFR immunoliposomes exhibit increased cytotoxicity on target cells. Eur. J. Pharm. Biopharm..

[28] Nassir A.M., Ibrahim I.A.A., Md S., Waris M., Ain M.R., Ahmad I., Shahzad N. (2019). Surface functionalized folate targeted oleuropein nano-liposomes for prostate tumor targeting: In vitro and in vivo activity. Life Sci..

[29] Yoon H.Y., Chang I.H., Goo Y.T., Kim C.H., Kang T.H., Kim S.Y., Lee S.J., Song S.H., Whang Y.M., Choi Y.W. (2019). Intravesical delivery of rapamycin via folate-modified liposomes dispersed in thermo-reversible hydrogel. Int. J. Nanomed..

[30] Akhtar A., Ghali L., Wang S.X., Bell C., Li D., Wen X. (2019). Optimisation of folate-mediated liposomal encapsulated arsenic trioxide for treating HPV-positive cervical cancer cells in vitro. Int. J. Mol. Sci..

[31] Sakpakdeejaroen I., Somani S., Laskar P., Mullin M., Dufès C. (2019). Transferrin-bearing liposomes entrapping plumbagin for targeted cancer therapy. J. Interdiscip. Nanomed..

[32] Jhaveri A., Deshpande P., Pattni B., Torchilin V. (2018). Transferrin-targeted, resveratrol-loaded liposomes for the treatment of glioblastoma. J. Control. Release.

[33] Ye J., Yang Y., Jin J., Ji M., Gao Y., Feng Y., Wang H., Chen X., Liu Y. (2020). Targeted delivery of chlorogenic acid by mannosylated liposomes to effectively promote the polarization of TAMs for the treatment of glioblastoma. Bioact. Mater..

[34] Vakhshiteh F., Khabazian E., Atyabi F., Ostad S.N., Madjd Z., Dinarvand R. (2020). Peptide-conjugated liposomes for targeted miR-34a delivery to suppress breast cancer and cancer stem-like population. J. Drug Deliv. Sci. Technol..

[35] Wang X., Zhao Y., Dong S., Lee R.J., Yang D., Zhang H., Teng L. (2019). Cell-penetrating peptide and transferrin co-modified liposomes for targeted therapy of glioma. Molecules.

[36] Lin Y.L., Tsai N.M., Chen C.H., Liu Y.K., Lee C.J., Chan Y.L., Wang Y.S., Chang Y.C., Lin C.H., Huang T.H. (2019). Specific drug delivery efficiently induced human breast tumor regression using a lipoplex by non-covalent association with anti-tumor antibodies. J. Nanobiotechnol..

[37] Yu S., Bi X., Yang L., Wu S., Yu Y., Jiang B., Zhang A., Lan K., Duan S. (2019). Co-delivery of paclitaxel and PLK1-targeted siRNA using aptamer-functionalized cationic liposome for synergistic anti-breast cancer effects in vivo. J. Biomed. Nanotechnol..

[38] Riaz M.K., Riaz M.A., Zhang X., Lin C., Wong K.H., Chen X., Zhang G., Lu A., Yang Z. (2018). Surface functionalization and targeting strategies of liposomes in solid tumor therapy: A review. Int. J. Mol. Sci..

[39] Nazeer N., Panicker J.T., Rajalekshmi S.M., Shaiju S.D.A. (2019). A Review on Surface Modified Sterically Stabilized Liposomes. Int. J. Innov. Sci. Res. Technol..

[40] Rowe R.C. (2020). Handbook of Pharmaceutical Excipients.

[41] Allen C.D.S.N., Dos Santos N., Gallagher R., Chiu G.N.C., Shu Y., Li W.M., Johnstone S.A., Janoff A.S., Mayer L.D., Webb M.S. (2002). Controlling the physical behavior and biological performance of liposome formulations through use of surface grafted poly (ethylene glycol). Biosci. Rep..

[42] Suk J.S., Xu Q., Kim N., Hanes J., Ensign L.M. (2016). PEGylation as a strategy for improving nanoparticle-based drug and gene delivery. Adv. Drug Deliv. Rev..

[43] Hatakeyama H., Akita H., Harashima H. (2013). The polyethyleneglycol dilemma: Advantage and disadvantage of PEGylation of liposomes for systemic genes and nucleic acids delivery to tumors. Biol. Pharm. Bull..

[44] AlSawaftah N., Pitt W.G., Husseini G.A. (2021). Dual-targeting and stimuli-triggered liposomal drug delivery in cancer treatment. ACS Pharmacol. Transl. Sci..

[45] Jain A., Jain S.K. (2018). Advances in tumor targeted liposomes. Curr. Mol. Med..

[46] Zhou J., Rossi J.J. (2011). Cell-specific aptamer-mediated targeted drug delivery. Oligonucleotides.

[47] Tuerk C., Gold L. (1990). Systematic evolution of ligands by exponential enrichment: RNA ligands to bacteriophage T4 DNA polymerase. Science.

[48] Derycke A.S., De Witte P.A. (2002). Transferrin-mediated targeting of hypericin embedded in sterically stabilized PEG-liposomes. Int. J. Oncol..

[49] Derycke A.S., Kamuhabwa A., Gijsens A., Roskams T., De Vos D., Kasran A., Huwyler J., Missiaen L., de Witte P.A. (2004). Transferrin- conjugated liposome targeting of photosensitizer AlPcS4 to rat bladder carcinoma cells. J. Natl. Cancer Inst..

[50] Hilgenbrink A.R., Low P.S. (2005). Folate receptor-mediated drug targeting: From therapeutics to diagnostics. J. Pharm. Sci..

[51] Paulos C.M., Reddy J.A., Leamon C.P., Turk M.J., Low P.S. (2004). Ligand binding and kinetics of folate receptor recycling in vivo: Impact on receptor-mediated drug delivery. Mol. Pharmacol..

[52] Sofou S., Sgouros G. (2008). Antibody-targeted liposomes in cancer therapy and imaging. Expert Opin Drug Deliv..

[53] Ichikawa K., Hikita T., Maeda N., Yonezawa S., Takeuchi Y., Asai T., Namba Y., Oku N. (2005). Antiangiogenic photodynamic therapy (PDT) by using long-circulating liposomes modified with peptide specific to angiogenic vessels. Biochim. Biophys. Acta Biomembr..

[54] Meng S., Su B., Li W., Ding Y., Tang L., Zhou W., Song Y., Li H., Zhou C. (2010). Enhanced antitumor effect of novel dual targeted paclitaxel liposomes. Nanotechnology.

[55] Saul J.M., Annapragada A.V., Bellamkonda R.V. (2006). A dual-ligand approach for enhancing targeting selectivity of therapeutic nanocarriers. J. Control. Release.

[56] Zhu Y., Feijen J., Zhong Z. (2018). Dual-targeted nanomedicines for enhanced tumor treatment. Nano Today.

[57] Mei L., Zhang Q., Yang Y., He Q., Gao H. (2014). Angiopep-2 and activatable cell penetrating peptide dual modified nanoparticles for enhanced tumor targeting and penetrating. Int. J. Pharm..

[58] Li H., Tsui T.Y., Ma W. (2015). Intracellular delivery of molecular cargo using cell-penetrating peptides and the combination strategies. Int. J. Mol. Sci..

[59] Huang Y., Jiang Y., Wang H., Wang J., Shin M.C., Byun Y., He H., Liang Y., Yang V.C. (2013). Curb challenges of the “Trojan Horse” approach: Smart strategies in achieving effective yet safe cell-penetrating peptide-based drug delivery. Adv. Drug Deliv. Rev..

[60] Gao H., Zhang Q., Yu Z., He Q. (2014). Cell-penetrating peptide-based intelligent liposomal systems for enhanced drug delivery. Curr. Pharm. Biotechnol..

[61] Desale K., Kuche K., Jain S. (2021). Cell-penetrating peptides (CPPs): An overview of applications for improving the potential of nanotherapeutics. Biomater. Sci..

[62] Zangabad P.S., Mirkiani S., Shahsavari S., Masoudi B., Masroor M., Hamed H., Jafari Z., Taghipour Y.D., Hashemi H., Karimi M. (2018). Stimulus-Responsive Liposomes as Smart Nanoplatforms for Drug Delivery Applications. Nanotechnol. Rev..

[63] Lee Y., Thompson D.H. (2017). Stimuli-Responsive Liposomes for Drug Delivery. Wiley Interdiscip. Rev. Nanomed. Nanobiotechnol..

[64] Tao Y., Chan H.F., Shi B., Li M., Leong K.W. (2020). Light: A magical tool for controlled drug delivery. Adv. Funct. Mater..

[65] Municoy S., Álvarez Echazú M.I., Antezana P.E., Galdopórpora J.M., Olivetti C., Mebert A.M., Foglia M.L., Tuttolomondo M.V., Alvarez G.S., Hardy J.G. (2020). Stimuli-responsive materials for tissue engineering and drug delivery. Int. J. Mol. Sci..

[66] Linsley C.S., Wu B.M. (2017). Recent advances in light-responsive on-demand drug-delivery systems. Ther. Deliv..

[67] Rapp T.L., DeForest C.A. (2021). Targeting drug delivery with light: A highly focused approach. Adv. Drug Deliv. Rev..

[68] Zhao W., Zhao Y., Wang Q., Liu T., Sun J., Zhang R. (2019). Remote light-responsive nanocarriers for controlled drug delivery: Advances and perspectives. Small.

[69] Seynhaeve A.L.B., Amin M., Haemmerich D., Van Rhoon G.C., Ten Hagen T.L.M. (2020). Hyperthermia and smart drug delivery systems for solid tumor therapy. Adv. Drug Deliv. Rev..

[70] Otto D.P., de Villiers M.M. (2014). What is the future of heated transdermal delivery systems?. Ther. Deliv..

[71] Banerjee R. (2011). Trigger-responsive nanoparticles: Control switches for cancer therapy. Nanomedicine.

[72] Schmaljohann D. (2006). Thermo-and pH-responsive polymers in drug delivery. Adv. Drug Deliv. Rev..

[73] Mura S., Nicolas J., Couvreur P. (2013). Stimuli-responsive nanocarriers for drug delivery. Nat. Mater..

[74] Wahajuddin S.A. (2012). Superparamagnetic iron oxide nanoparticles: Magnetic nanoplatforms as drug carriers. Int. J. Nanomed..

[75] Liu J.F., Jang B., Issadore D., Tsourkas A. (2019). Use of magnetic fields and nanoparticles to trigger drug release and improve tumor targeting. Wiley Interdiscip. Rev. Nanomed. Nanobiotechnol..

[76] Anderson S.D., Gwenin V.V., Gwenin C.D. (2019). Magnetic functionalized nanoparticles for biomedical, drug delivery and imaging applications. Nanoscale Res. Lett..

[77] Cai X., Jiang Y., Lin M., Zhang J., Guo H., Yang F., Leung W., Xu C. (2020). Ultrasound-responsive materials for drug/gene delivery. Front. Pharmacol..

[78] Mehier-Humbert S., Bettinger T., Yan F., Guy R.H. (2005). Plasma membrane poration induced by ultrasound exposure: Implication for drug delivery. J. Control. Release.

[79] Leung S.J., Romanowski M. (2012). Light-activated content release from liposomes. Theranostics.

[80] Goulet-Hanssens A., Eisenreich F., Hecht S. (2020). Enlightening materials with photoswitches. Adv. Mater..

[81] Russew M.M., Hecht S. (2010). Photoswitches: From molecules to materials. Adv. Mater..

[82] Liu R., Zhang X., Xia F., Dai Y. (2022). Azobenzene-based photoswitchable catalysts: State of the art and perspectives. J. Catal..

[83] Sponza A.D., Liu D., Chen E.P., Shaw A., Diawara L., Chiu M. (2020). Synthesis strategies for non-symmetric, photochromic diarylethenes. Org. Biomol. Chem..

[84] Kortekaas L., Browne W.R. (2019). The evolution of spiropyran: Fundamentals and progress of an extraordinarily versatile photochrome. Chem Soc. Rev..

[85] Liu Y., An X. (2019). Preparation, microstructure and function of liposome with light responsive switch. Colloids Surf. B.

[86] Zhang D., Shah P.K., Culver H.R., David S.N., Stansbury J.W., Yin X., Bowman C.N. (2019). Photo-responsive liposomes composed of spiropyran-containing triazole-phosphatidylcholine: Investigation of merocyanine-stacking effects on liposome–fiber assembly-transition. Soft Matter.

[87] Liu J.X., Xin B., Li C., Xie N.H., Gong W.L., Huang Z.L., Zhu M.Q. (2017). PEGylated perylenemonoimide-dithienylethene for super-resolution imaging of liposomes. ACS Appl. Mater. Interfaces.

[88] Dariva C.G., Coelho J.F., Serra A.C. (2019). Near infrared light-triggered nanoparticles using singlet oxygen photocleavage for drug delivery systems. J. Control. Release.

[89] Franco M.S., Gomes E.R., Roque M.C., Oliveira M.C. (2021). Triggered drug release from liposomes: Exploiting the outer and inner tumor environment. Front. Oncol..

[90] Hrycay E.G., Bandiera S.M. (2015). Involvement of cytochrome P450 in reactive oxygen species formation and cancer. Adv. Pharmacol..

[91] Correia J.H., Rodrigues J.A., Pimenta S., Dong T., Yang Z. (2021). Photodynamic therapy review: Principles, photosensitizers, applications, and future directions. Pharmaceutics.

[92] Plaetzer K., Krammer B., Berlanda J., Berr F., Kiesslich T. (2009). Photophysics and photochemistry of photodynamic therapy: Fundamental aspects. Lasers Med. Sci..

[93] Kudinova N.V., Berezov T.T. (2010). Photodynamic therapy of cancer: Search for ideal photosensitizer. Biochemistry.

[94] O’Connor A.E., Gallagher W.M., Byrne A.T. (2009). Porphyrin and nonporphyrin photosensitizers in oncology: Preclinical and clinical advances in photodynamic therapy. Photochem. Photobiol..

[95] Sun B., Ghosh S., He X., Huang W.C., Quinn B., Tian M., Jahagirdar D., Mabrouk M.T., Ortega J., Zhang Y. (2022). Anti-cancer liposomal chemophototherapy using bilayer-localized photosensitizer and cabazitaxel. Nano Res..

[96] Lovell J.F., Jin C.S., Huynh E., Jin H., Kim C., Rubinstein J.L., Chan W.C.W., Cao W., Wang L.V., Zheng G. (2011). Porphysome nanovesicles generated by porphyrin bilayers for use as multimodal biophotonic contrast agents. Nat. Mater..

[97] Carter K.A., Shao S., Hoopes M.I., Luo D., Ahsan B., Grigoryants V.M., Song W., Huang H., Zhang G., Pandey R.K. (2014). Porphyrin-phospholipid liposomes permeabilized by near-infrared light. Nat. Commun..

[98] Massiot J., Rosilio V., Makky A. (2019). Photo-triggerable liposomal drug delivery systems: From simple porphyrin insertion in the lipid bilayer towards supramolecular assemblies of lipid–porphyrin conjugates. J. Mater. Chem. B.

[99] Xue X., Lindstrom A., Li Y. (2019). Porphyrin-based nanomedicines for cancer treatment. Bioconjug. Chem..

[100] Cressey P., Bronstein L.G., Benmahmoudi R., Rosilio V., Regeard C., Makky A. (2022). Novel liposome-like assemblies composed of phospholipid-porphyrin conjugates with photothermal and photodynamic activities against bacterial biofilms. Int. J. Pharm..

[101] Massiot J., Abuillan W., Konovalov O., Makky A. (2022). Photo-triggerable liposomes based on lipid-porphyrin conjugate and cholesterol combination: Formulation and mechanistic study on monolayers and bilayers. Biochim. Biophys. Acta Biomembr..

[102] Huang X., El-Sayed M.A. (2010). Gold nanoparticles: Optical properties and implementations in cancer diagnosis and photothermal therapy. J. Adv. Res..

[103] Salkho N.M., Awad N.S., Pitt W.G., Husseini G.A. (2022). Photo-induced drug release from polymeric micelles and liposomes: Phototriggering mechanisms in drug delivery systems. Polymers.

[104] Rubio-Camacho M., Martínez-Tomé M.J., Cuestas-Ayllón C., de la Fuente J.M., Esquembre R., Mateo C.R. (2023). Tailoring the plasmonic properties of gold-liposome nanohybrids as a potential powerful tool for light-mediated therapies. Colloids Interface Sci. Commun..

[105] Liu Y., He M., Niu M., Zhao Y., Zhu Y., Li Z., Feng N. (2015). Delivery of vincristine sulfate-conjugated gold nanoparticles using liposomes: A light-responsive nanocarrier with enhanced antitumor efficiency. Int. J. Nanomed..

[106] Fomina N., Sankaranarayanan J., Almutairi A. (2012). Photochemical mechanisms of light-triggered release from nanocarriers. Adv. Drug Deliv. Rev..

[107] Zhao X., Fang X., Yang S., Zhang S., Yu G., Liu Y., Zhou Y., Feng Y., Li J. (2021). Light-tuning amphiphility of host-guest Alginate-based supramolecular assemblies for photo-responsive Pickering emulsions. Carbohydr. Polym..

[108] Rideau E., Dimova R., Schwille P., Wurm F.R., Landfester K. (2018). Liposomes and polymersomes: A comparative review towards cell mimicking. Chem. Soc. Rev..

[109] Leong J., Teo J.Y., Aakalu V.K., Yang Y.Y., Kong H. (2018). Engineering Polymersomes for Diagnostics and Therapy. Adv. Healthc. Mater..

[110] Yamamoto S., Yamada T., Kubo G., Sakurai K., Yamaguchi K., Nakanishi J. (2019). Preparation of a series of photoresponsive polymersomes bearing photocleavable a 2-nitrobenzyl group at the hydrophobic/hydrophilic interfaces and their payload releasing behaviors. Polymers.

[111] Regen S.L., Singh A., Oehme G., Singh M. (1981). Polymerized phosphatidylcholine vesicles. Stabilized and controllable time-release carriers. Biochem. Biophys. Res. Commun..

[112] Nakamura S., Uehara H., Hasegawa T., Fujimoto K. (2017). Phototriggered Sequence-specific DNA Transportation into Liposomes Using Ultrafast DNA Photocrosslinking. Chem. Lett..

[113] He J., Tong X., Zhao Y. (2009). Photoresponsive nanogels based on photocontrollable cross-links. Macromolecules.

[114] Lu D., Zhu M., Wu S., Wang W., Lian Q., Saunders B.R. (2019). Triply responsive coumarin-based microgels with remarkably large photo-switchable swelling. Polym. Chem..

[115] Heidarli E., Dadashzadeh S., Haeri A. (2017). State of the art of stimuli-responsive liposomes for cancer therapy. Iran. J. Pharm. Res..

[116] Raza A., Rasheed T., Nabeel F., Hayat U., Bilal M., Iqbal H.M.N. (2019). Endogenous and exogenous stimuli-responsive drug delivery systems for programmed site-specific release. Molecules.

[117] Li H., Yang X., Zhou Z., Wang K., Li C., Qiao H., Oupicky D., Sun M. (2017). Near-Infrared light-triggered drug release from a multiple lipid carrier complex using an all-in one strategy. J. Controll. Release.

[118] Refaat A., Del Rosal B., Palasubramaniam J., Pietersz G., Wang X., Moulton S.E., Peter K. (2021). Near-infrared light-responsive liposomes for protein delivery: Towards bleeding-free *photothermally*-assisted thrombolysis. J. Control. Release.

[119] Yang G., Liu J., Wu Y., Feng L., Liu Z. (2016). Near-Infrared-Light responsive nanoscale drug delivery systems for cancer treatment. Coord. Chem. Rev..

[120] Zeng X.L., Zhou X.C., Wu S. (2018). Red and near-infrared light-cleavable polymers. Macromol. Rapid Commun..

[121] Zhu X.J., Su Q.Q., Feng W., Li F.Y. (2017). Anti-Stokes shift luminescent materials for bio-applications. Chem. Soc. Rev..

[122] Sun Y., Ji Y., Yu H., Wang D., Cao M., Wang J. (2016). Near-infrared light-sensitive liposomes for controlled release. RSC Adv..

[123] Gwon K., Jo E.-J., Sahu A., Lee J.Y., Kim M.-G., Tae G. (2018). Improved near infrared-mediated hydrogel formation using diacrylated Pluronic F127-Coated upconversion nanoparticles. Mater. Sci. Eng. C.

[124] Wu S., Butt H.J. (2016). Near-infrared-sensitive materials based on upconverting nanoparticles. Adv. Mater..

[125] Wen S.H., Zhou J.J., Zheng K.Z., Bednarkiewicz A., Liu X.G., Jin D.Y. (2018). Advances in highly doped upconversion nanoparticles. Nat. Commun..

[126] Xiang J., Tong X., Shi F., Yan Q., Yu B., Zhao Y. (2018). Near-Infrared light-triggered drug release from UV-responsive di-block copolymer-coated upconversion nanoparticles with high monodispersity. J. Mater. Chem. B.

[127] Yi C., Yu Z., Ren Q., Liu X., Wang Y., Sun X., Yin S., Pan J., Huang X. (2020). Nanoscale ZnO-Based photosensitizers for photodynamic therapy. Photodiagnosis Photodyn. Ther..

[128] Li Q., Li W., Di H., Luo L., Zhu C., Yang J., Yin X., Yin H., Gao J., Du Y. (2018). A photosensitive liposome with NIR light triggered doxorubicin release as a combined photodynamic-chemo therapy system. J. Control. Release.

[129] Li Y., Zhang Y., Wang W. (2018). Phototriggered targeting of nanocarriers for drug delivery. Nano Res..

[130] Xie X., Yang Y., Yang Y., Mei X. (2015). Photolabile-caged peptide-conjugated liposomes for siRNA delivery. J. Drug Target..

[131] Xie X., Yang Y., Yang Y., Zhang H., Li Y., Mei X. (2016). A photo-responsive peptide-and asparagine–glycine–arginine (NGR) peptide-mediated liposomal delivery system. Drug Deliv..

[132] Hansen M.B., Van Gaal E., Minten I., Storm G., Van Hest J.C., Löwik D.W. (2012). Constrained and UV-activatable cell-penetrating peptides for intracellular delivery of liposomes. J. Control. Release.

[133] Yang Y., Yang Y., Xie X., Wang Z., Gong W., Zhang H., Li Y., Yu F., Li Z., Mei X. (2015). Dual-modified liposomes with a two-photon-sensitive cell penetrating peptide and NGR ligand for siRNA targeting delivery. Biomaterials.

[134] Mizukami S., Hosoda M., Satake T., Okada S., Hori Y., Furuta T., Kikuchi K. (2010). Photocontrolled compound release system using caged antimicrobial peptide. J. Am. Chem. Soc..

[135] Fan N.C., Cheng F.Y., Ho J.A.A., Yeh C.S. (2012). Photocontrolled targeted drug delivery: Photocaged biologically active folic acid as a light-responsive tumor-targeting molecule. Angew. Chem..

[136] He C., Hu Y., Yin L., Tang C., Yin C. (2010). Effects of particle size and surface charge on cellular uptake and biodistribution of polymeric nanoparticles. Biomaterials.

[137] Tong R., Chiang H.H., Kohane D.S. (2013). Photoswitchable nanoparticles for in vivo cancer chemotherapy. Proc. Natl. Acad. Sci. USA.

[138] Cabral H., Matsumoto Y., Mizuno K., Chen Q., Murakami M., Kimura M., Terada Y., Kano M.R., Miyazono K., Uesaka M.J.N.N. (2011). Accumulation of sub-100 nm polymeric micelles in poorly permeable tumours depends on size. Nat. Nanotechnol..

[139] Qiu L., Chen T., Öçsoy I., Yasun E., Wu C., Zhu G., You M., Han D., Jiang J., Yu R. (2015). A cell-targeted, size-photocontrollable, nuclear-uptake nanodrug delivery system for drug-resistant cancer therapy. Nano Lett..

[140] Ojha T., Pathak V., Shi Y., Hennink W.E., Moonen C.T., Storm G., Kiessling F., Lammers T. (2017). Pharmacological and physical vessel modulation strategies to improve EPR-mediated drug targeting to tumors. Adv. Drug Deliv. Rev..

[141] Tour O., Meijer R.M., Zacharias D.A., Adams S.R., Tsien R.Y. (2003). Genetically targeted chromophore-assisted light inactivation. Nat. Biotechnol..

[142] Agostinis P., Berg K., Cengel K.A., Foster T.H., Girotti A.W., Gollnick S.O., Hahn S.M., Hamblin M.R., Juzeniene A., Kessel D. (2011). Photodynamic therapy of cancer: An update. CA Cancer J. Clin..

[143] Mitsunaga M., Ogawa M., Kosaka N., Rosenblum L.T., Choyke P.L., Kobayashi H. (2011). Cancer cell–selective in vivo near infrared photoimmunotherapy targeting specific membrane molecules. Nat. Med..

[144] Sano K., Nakajima T., Choyke P.L., Kobayashi H. (2013). Markedly enhanced permeability and retention effects induced by photo-immunotherapy of tumors. ACS Nano.

[145] Puri A. (2014). Phototriggerable liposomes: Current research and future perspectives. Pharmaceutics.

[146] Miranda D., Lovell J.F. (2016). Mechanisms of light-induced liposome permeabilization. Bioeng. Transl. Med..

[147] Mathiyazhakan M., Wiraja C., Xu C. (2018). A concise review of gold nanoparticles-based photo-responsive liposomes for controlled drug delivery. Nanomicro Lett..

[148] Paasonen L., Sipilä T., Subrizi A., Laurinmäki P., Butcher S.J., Rappolt M., Yaghmur A., Urtti A., Yliperttula M. (2010). Gold-embedded photosensitive liposomes for drug delivery: Triggering mechanism and intracellular release. J. Control. Release.

[149] Yue H., Wei W., Yue Z., Lv P., Wang L., Ma G., Su Z. (2010). Particle size affects the cellular response in macrophages. Eur. J. Pharm. Sci..

[150] Jasinski D.L., Li H., Guo P. (2018). The effect of size and shape of RNA nanoparticles on biodistribution. Mol. Ther..

[151] Hardonk M.J., Harms G., Koudstaal J. (1985). Zonal heterogeneity of rat hepatocytes in the in vivo uptake of 17nm colloidal gold granules. Histochemistry.

[152] Di J., Gao X., Du Y., Zhang H., Gao J., Zheng A. (2021). Size, shape, charge and “stealthy” surface: Carrier properties affect the drug circulation time in vivo. Asian J. Pharm. Sci..

[153] Woodle M.C., Papahadjopoulos D. (1989). Liposome preparation and size characterization. Methods Enzymol..

[154] Kulkarni S.B., Betageri G.V., Singh M. (1995). Factors affecting microencapsulation of drugs in liposomes. J. Microencapsul..

[155] Perkins W.R., Minchey S.R., Ahl P.L., Janoff A.S. (1993). The determination of liposome captured volume. Chem. Phys. Lipids..

[156] Filipczak N., Pan J., Yalamarty S.S.K., Torchilin V.P. (2020). Recent advancements in liposome technology. Adv. Drug Deliv. Rev..

[157] Myint K.T., Sahoo S., Thein A.W., Moe S., Ni H. (2022). Laser therapy for retinopathy in sickle cell disease. Cochrane Database Syst. Rev..

[158] Jelínková H. (2013). Lasers for Medical Applications: Diagnostics, Therapy and Surgery.

[159] Bastos J., Lizarelli R., Parizotto N. (2009). Comparative study of laser and LED systems of low intensity applied to tendon healing. Laser Phys..

[160] Tong R., Kohane D.S. (2012). Shedding light on nanomedicine. Wiley Interdiscip. Rev. Nanomed. Nanobiotechnol..

[161] Rwei A.Y., Wang W., Kohane D.S. (2015). Photoresponsive nanoparticles for drug delivery. Nano Today.

[162] Barat K. (2008). Laser Safety: Tools and Training.

[163] Ash C., Dubec M., Donne K., Bashford T. (2017). Effect of wavelength and beam width on penetration in light-tissue interaction using computational methods. Lasers Med Sci..

[164] Gunaydin G., Gedik M.E., Ayan S. (2021). Photodynamic therapy for the treatment and diagnosis of cancer—A review of the current clinical status. Front. Chem..

[165] Gonzaga E.R. (2009). Role of UV light in photodamage, skin aging, and skin cancer: Importance of photoprotection. Am. J. Clin. Dermatol..

[166] Organisciak D.T., Vaughan D.K. (2010). Retinal light damage: Mechanisms and protection. Prog. Retin. Eye Res..

[167] Ji Y., Jones C., Baek Y., Park G.K., Kashiwagi S., Choi H.S. (2020). Near-infrared fluorescence imaging in immunotherapy. Adv. Drug Deliv. Rev..

[168] Mi P. (2020). Stimuli-responsive nanocarriers for drug delivery, tumor imaging, therapy and theranostics. Theranostics.

[169] Kong F., Zhang H., Zhang X., Liu D., Chen D., Zhang W., Zhang L., Santos H.A., Hai M. (2016). Biodegradable photothermal and pH responsive calcium carbonate@ phospholipid@ acetalated dextran hybrid platform for advancing biomedical applications. Adv. Funct. Mater..

[170] Chen M.M., Song F.F., Feng M., Liu Y., Liu Y.Y., Tian J., Lv F., Zhang Q.Q. (2018). pH-sensitive charge-conversional and NIR responsive bubble-generating liposomal system for synergetic thermo-chemotherapy. Colloids Surf. B.

[171] You C., Wang M., Wu H., An P., Pan M., Luo Y., Sun B. (2017). Near infrared radiated stimulus-responsive liposomes based on photothermal conversion as drug carriers for co-delivery of CJM126 and cisplatin. Mater. Sci. Eng. C.

[172] Luo L., Bian Y., Liu Y., Zhang X., Wang M., Xing S., Li L., Gao D. (2016). Combined near infrared photothermal therapy and chemotherapy using gold nanoshells coated liposomes to enhance antitumor effect. Small.

[173] Lin H.C., Li W.T., Madanayake T.W., Tao C., Niu Q., Yan S.Q., Gao B.A., Ping Z. (2020). Aptamer-guided upconversion nanoplatform for targeted drug delivery and near-infrared light-triggered photodynamic therapy. J. Biomater. Appl..

[174] Tian M., Xin X., Wu R., Guan W., Zhou W. (2022). Advances in intelligent-responsive nanocarriers for cancer therapy. Pharmacol. Res..

[175] Chamundeeswari M., Jeslin J., Verma M.L. (2019). Nanocarriers for drug delivery applications. Environ. Chem. Lett..

[176] Fournier L., Gauron C., Xu L., Aujard I., Le Saux T., Gagey-Eilstein N., Maurin S., Dubruille S., Baudin J.B., Bensimon D. (2013). A blue-absorbing photolabile protecting group for in vivo chromatically orthogonal photoactivation. ACS Chem. Biol..

[177] Olson J.P., Kwon H.B., Takasaki K.T., Chiu C.Q., Higley M.J., Sabatini B.L., Ellis-Davies G.C. (2013). Optically selective two-photon uncaging of glutamate at 900 nm. J. Am. Chem. Soc..

[178] Fournier L., Aujard I., Le Saux T., Maurin S., Beaupierre S., Baudin J.B., Jullien L. (2013). Coumarinylmethyl caging groups with redshifted absorption. Chem. Eur. J..

[179] Gandioso A., Cano M., Massaguer A., Marchán V. (2016). A green light-triggerable RGD peptide for photocontrolled targeted drug delivery: Synthesis and photolysis studies. J. Org. Chem..

[180] Huang L., Zhao Y., Zhang H., Huang K., Yang J., Han G. (2017). Expanding anti-Stokes shifting in triplet–triplet annihilation upconversion for in vivo anticancer prodrug activation. Angew. Chem..

[181] Li D., Ma Y., Du J., Tao W., Du X., Yang X., Wang J. (2017). Tumor acidity/NIR controlled interaction of transformable nanoparticle with biological systems for cancer therapy. Nano Lett..

[182] Lin Q., Bao C., Yang Y., Liang Q., Zhang D., Cheng S., Zhu L. (2013). Highly discriminating photorelease of anticancer drugs based on hypoxia activatable phototrigger conjugated chitosan nanoparticles. Adv. Mater..

[183] Nikolova M.P., Kumar E.M., Chavali M.S. (2022). Updates on Responsive Drug Delivery Based on Liposome Vehicles for Cancer Treatment. Pharmaceutics.

[184] Nsairat H., AlShaer W., Odeh F., Essawi E., Khater D., Al Bawab A., El-Tanani M., Awidi A., Mubarak M.S. (2023). Recent Advances in Using Liposomes for Delivery of Nucleic Acid-Based Therapeutics. OpenNano.

[185] Magar K.T., Boafo G.F., Li X., Chen Z., He W. (2022). Liposome-based delivery of biological drugs. Chin. Chem. Lett..

